# Transversal modes and Higgs bosons in electroweak vector-boson scattering at the LHC

**DOI:** 10.1140/epjc/s10052-018-6398-4

**Published:** 2018-11-14

**Authors:** Simon Brass, Christian Fleper, Wolfgang Kilian, Jürgen Reuter, Marco Sekulla

**Affiliations:** 1 Department of Physics, University of Siegen, 57068 Siegen, Germany; 2 CLICdp and Theory Group, CERN, 1211 Geneva 23, Switzerland; 3 DESY Theory Group, 22607 Hamburg, Germany; 4 Institute for Theoretical Physics, Karlsruhe Institute of Technology, 76128 Karlsruhe, Germany

## Abstract

Processes where *W* and *Z* bosons scatter into pairs of electroweak bosons *W*, *Z*, and Higgs, are sensitive probes of new physics in the electroweak sector. We study simplified models that describe typical scenarios of new physics and parameterize the range of possible LHC results between the standard-model prediction and unitarity limits. Extending the study beyond purely longitudinal scattering, we investigate the role of transversally polarized gauge bosons. Unitarity becomes an essential factor, and limits on parameters matched to the naive perturbative low-energy effective theory turn out to be necessarily model-dependent. We discuss the implications of our approach for the interpretation of LHC data on vector-boson scattering and Higgs-pair production.

## Introduction

After the discovery of the Higgs boson [1, 2], particle physics faces the question whether the new scalar sector is minimal or non-minimal, whether it is weakly or strongly interacting, and whether it validates or invalidates the accepted paradigm of quantum field theory as a universal description of particle interactions. Experimental data on electroweak boson interactions (Higgs, *W*, *Z*, and photon) will deepen our understanding in that area. Processes of the type $VV\rightarrow VV$, where $V=W,Z,H$ (vector-boson scattering, VBS, and associated or Higgs pair production in vector-boson fusion, VBF), are a most sensitive probe of electroweak physics and the Higgs sector. They will be extensively studied in the present and future runs of the LHC.

There is obvious interest in scenarios where new degrees of freedom beyond the standard model (SM) couple primarily to the Higgs-Goldstone boson field. The presence of new physics coupled to the Higgs sector might solve some of the long-standing puzzles of particle physics. Such new modes need not have a significant effect on existing precision data. They might be strongly-interacting as in composite Higgs models, or weakly interacting as in models with extra scalars that are decoupled from SM fermions. They should manifest themselves primarily in interactions of massive electroweak bosons, namely $W^\pm$, *Z*, and the Higgs itself.

The ATLAS and CMS experiments at the LHC have measured VBS processes as a signal, embedded in partonic processes of the type $qq\rightarrow VVqq$, where *q* is any light quark. Numerical results have been presented in the form of limits on parameters within the SM effective theory (SMEFT) [3–6]. The usual application of the SMEFT truncates the power expansion of the Lagrangian at the level of dimension-six operators. A useful parameterization of VBS processes requires dimension-eight effective operators, the second order of the low-energy expansion beyond the SM.

In recent work [7, 9, 10], we have studied deviations from the SM interactions that are confined to the longitudinal scattering modes of *W* and *Z*. In the low-energy limit, the contributing set of new interactions is small, and if custodial (weak isospin) symmetry is imposed, there are just two free parameters in the matching SMEFT expansion. On the other hand, recent LHC analyses quote results for a larger set of operator coefficients which include interactions between longitudinal and transversal modes of *W* and *Z* bosons. In the current paper, we study deviations from the SM in VBS processes that involve transversally polarized *W* and *Z* bosons, and also consider Higgs bosons in the final state.

Numerical results of non-SM interactions of longitudinal scattering have clearly shown that for the level of deviations that can be detected by the LHC experiments, the unitarity limits are always violated in the high-energy range, if a naive SMEFT calculation is attempted. A model-independent parameterization beyond the SM that covers the accessible parameter range becomes impossible. Nevertheless, reasonable assumptions on new physics lead to unitarity constraints that limit the level of possible excess above the SM prediction. With this knowledge, it is possible to devise simplified models that both satisfy unitarity over the whole energy range and smoothly match onto the SMEFT parameterization at low energy. For the purpose of an exemplary study, we have compared a class of “continuum” models which merely extrapolate the SMEFT expansion into asymptotically strong interactions with models that describe single resonances with specific quantum-number assignments.

In the present paper, we extend this program to also describe transversal modes, and to include the Higgs boson as a possible final state on the same footing as the *W* and *Z* bosons. Regarding the SM processes as reference, there are detailed calculations [11–17] beyond leading-order in the SM perturbation theory. Recently, there was a concise comparison of several different codes for the precision simulation at LO and NLO for like-sign vector boson scattering at 13 TeV [18]. For the simplified models considered in this paper, we confine ourselves to leading-order calculations but we remark that adding in perturbative QCD and electroweak corrections is possible by the same methods as for the pure SM, and should eventually be done in order to distinguish genuine deviations from uncertainties of the approximation, in actual data analysis.

The paper is organized as follows. In Sect. 2 we discuss the structure of new interactions in the electroweak and Higgs sector, and state the underlying assumptions. This defines the SMEFT ansatz, and it allows us to list the operators that describe the low-energy limit. The symmetries of those interactions determine the eigenmodes of quasi-elastic $2\rightarrow 2$ scattering, which allows us to diagonalize the amplitudes for all vector-boson modes, Sect. 3. We construct unitary models exhibiting a strongly interacting continuum in Sect. 4. These models would yield the maximally allowed number of events consistent with quantum field theory in the VBS channel, matched to the low-energy SMEFT with specific values for the operator coefficients. In Sect. 5 we discuss simplified models which contain a resonance and likewise parameterize VBS amplitudes at all energies. We present numerical results and plots for selected parameter sets and final states. In Sect. 6, we discuss the relevance of our study in view of future analyses at the LHC and beyond.

## Electroweak boson currents and local operators

Expectations for new physics beyond the SM are constrained by available data. They may also be guided by imposing principles such as simplicity and absence of accidental cancellations. For the current work, we base the description of new effects beyond the SM on the following assumptions: (i) light fermions do not participate directly in new dynamics, (ii) the observed pattern of SM gauge invariance retains its relevance beyond the $\text {TeV}$ range, and (iii) new degrees of freedom beyond the SM do not carry open color. These assumptions are not mandatory but backed by the available precision data regarding flavor physics, QCD, new-physics searches at the LHC, and precision electroweak observables.

If these assumptions are accepted as guiding principles for a phenomenological description, a parameterization of dominant new effects can qualify light-fermion currents as classical spectator fields, and focus on the bosonic SM multiplets acting as currents that probe the new sector. The currents can be introduced as local operators that couple to an unknown new-physics spectrum in a manifestly $SU(2)_L\times U(1)_Y$ invariant way. New dynamics may involve weakly coupled (comparatively) light degrees of freedom, such as extra Higgs singlets or doublets, it may probe a strongly coupled sector which is resolved at high energy, or it may give rise to heavy resonances, to name a few possibilities. In any case, new physics that is coupled to SM bosons, will manifest itself in anomalous scattering matrix elements of those bosons, and should become accessible in high-energy VBS. As a common feature of this class of models, we expect the scattering matrix to be self-contained and complete in terms of SM bosons and eventual new-physics states, to a good approximation.^1^

For a quantitative representation, we adopt the assumption of gauge invariance and describe new physics as coupled to gauge-covariant monomials of SM fields. For the building blocks, we introduce the Higgs multiplet in form of a $2\times 2$ matrix,

1 $$\begin{aligned} \mathbf {H}&= \frac{1}{2} \begin{pmatrix} v+ h -\mathrm {i}w^3 &{} -\mathrm {i}\sqrt{2} w^+ \\ -\mathrm {i}\sqrt{2} w^- &{} v + h + \mathrm {i}w^3 \\ \end{pmatrix}. \end{aligned}$$

The components $h,w^\pm ,w^3$ are the physical Higgs and unphysical Goldstone scalars, respectively, and *v* denotes the numerical Higgs vev, $v=246\;\text {GeV}$. The matrix notation allows us to manifestly represent the larger global symmetry on the Higgs field, $O(4)\sim SU(2)_L\times SU(2)_R$ which after electroweak symmetry breaking (EWSB) becomes the approximate custodial $SU(2)_c$ symmetry. $SU(2)_L\times SU(2)_R$-symmetric monomials are invariant under bi-unitary transformations of the form $\mathbf {H}\rightarrow U_L \mathbf {H}U_R^\dagger$.

The covariant derivative of the Higgs matrix is defined as

2 $$\begin{aligned} \mathbf {D}_\mu \mathbf {H}= \partial _\mu \mathbf {H}- \mathrm {i}g \mathbf {W}_\mu \mathbf {H}+ \mathrm {i}g^\prime \mathbf {H}\mathbf {B}_\mu , \end{aligned}$$

where

3 $$\begin{aligned} \mathbf {W}_\mu \equiv \mathbf {W}_\mu ^a \frac{\tau ^a}{2}, \qquad \mathbf {B}_\mu \equiv \mathbf {B}_\mu ^a \frac{\tau ^3}{2}. \end{aligned}$$

The transformation of $\mathbf {W}_\mu$ is $\mathbf {W}_\mu \rightarrow U_L^\dagger \mathbf {W}_\mu U_L$, while $\mathbf {B}_\mu$ transforms covariantly only under a $U(1)_R$ subgroup of $SU(2)_R$. The matrix-valued field strengths are given by

4 $$\begin{aligned}&\mathbf {W}_{\mu \nu } = \partial _\mu \mathbf {W}_\nu - \partial _\nu \mathbf {W}_\nu - i g \left[ \mathbf {W}_\mu , \mathbf {W}_\nu \right] , \nonumber \\&\mathbf {B}_{\mu \nu } = \partial _\mu \mathbf {B}_\nu - \partial _\nu \mathbf {B}_\nu . \end{aligned}$$

From these fields, we can build local composite operators which act as currents that probe the new, possibly non-local dynamics. For instance, the simplest Higgs-field currents are

5 $$\begin{aligned}&J_H^{(2)} = {\text {tr}}\left[ \mathbf {H}^\dagger \mathbf {H}\right] , \nonumber \\&J_H^{(4)} = {\text {tr}}\left[ (\mathbf {D}_\mu \mathbf {H})^\dagger (\mathbf {D}^\mu \mathbf {H})\right] \nonumber \\&J_{H\mu \nu }^{(4)}= {\text {tr}}\left[ (\mathbf {D}_\mu \mathbf {H})^\dagger (\mathbf {D}_\nu \mathbf {H})\right] , \end{aligned}$$

while gauge-field tensors can be combined as

6 $$\begin{aligned} J_W^{(4)}&= g^2{\text {tr}}\left[ \mathbf {W}_{\mu \nu }\mathbf {W}^{\mu \nu }\right] ,&J_B^{(4)}&= g'{}^2{\text {tr}}\left[ \mathbf {B}_{\mu \nu }\mathbf {B}^{\mu \nu }\right] , \end{aligned}$$

7 $$\begin{aligned} J_{W\mu \nu }^{(4)}&= g^2{\text {tr}}\left[ \mathbf {W}_{\mu \rho }\mathbf {W}^\rho _{\;\nu }\right] ,&J_{B\mu \nu }^{(4)}&= g'{}^2{\text {tr}}\left[ \mathbf {B}_{\mu \rho }\mathbf {B}^\rho _{\;\nu }\right] . \end{aligned}$$

These terms are electroweak singlets. Non-singlet currents can likewise be constructed.

We expect only weak constraints from existing data, so new dynamics, whether parameterized by form factors, spectral functions, or inelastic scattering into new particles, is rather arbitrary. For the purpose of this work, we focus on two extreme scenarios: (i) a spectrum that interpolates the low-energy description with unitarity saturation in the high-energy range, and (ii) a spectrum that consists of separate narrow to medium-width resonances, which we may reduce to the lowest-lying state for simplicity. For reference, we also include (iii) the unmodified SM where any new spectral functions are zero and all amplitudes remain weakly interacting. In terms of quasi-elastic $2\rightarrow 2$ scattering, scenarios (i) and (iii) correspond to maximal and minimal event yields in the asymptotic region, while (ii) exhibits unitarity saturation at finite energy. Another extreme scenario, saturation by inelastic scattering into new final states, asymptotically implies quasi-elastic event rates between (i) and (iii) and should furthermore be accessible via direct observation of new particles.

As a first step, we may confine the analysis to pure Higgs- and Goldstone-boson scattering. This was done in our earlier paper [7]. Such a restriction implies further assumptions on the underlying complete theory. In this work, we remove this restriction. We investigate the bosonic $2\rightarrow 2$ scattering matrix with Higgs, longitudinal, and transversal vector bosons included.

Unless there are undetected light particles hiding in this scattering matrix, it allows for a local operator-product expansion. Contracting the singlet currents listed above and ignoring terms which merely renormalize SM parameters, the leading terms are dimension-six operators: $(J_H^{(2)})^3$, $J_H^{(2)}J_H^{(4)}$, and $J_H^{(2)}J_W^{(4)}$. Only the latter term is easily accessible at the LHC,^2^ so a phenomenological parameterization should consider the next order of the expansion. These are dimension-eight local interactions.

Including all singlet and non-singlet operator products, and omitting CP-odd interactions, we can identify three distinct categories of dimension-eight bosonic operators in the low-energy expansion that we list below.

There are two terms which couple only Higgs-field currents,

8a $$\begin{aligned} {\mathcal {L}}_{S,0}&=F_{S,0}\text {tr}\left[ (\mathbf D _\mu \mathbf H )^\dagger (\mathbf D _\nu \mathbf H )\right] \text {tr}\left[ (\mathbf D ^\mu \mathbf H )^\dagger (\mathbf D ^\nu \mathbf H )\right] , \end{aligned}$$

8b $$\begin{aligned} {\mathcal {L}}_{S,1}&=F_{S,1}\text {tr}\left[ (\mathbf D _\mu \mathbf H )^\dagger (\mathbf D ^\mu \mathbf H )\right] \text {tr}\left[ (\mathbf D _\nu \mathbf H )^\dagger (\mathbf D ^\nu \mathbf H )\right] \; ; \end{aligned}$$

seven terms which couple Higgs- and gauge field currents,

9a $$\begin{aligned} {\mathcal {L}}_{M,0}&=-g^2 F_{M_0}\text {tr}\left[ (\mathbf D _\mu \mathbf H )^\dagger (\mathbf D ^\mu \mathbf H )\right] \text {tr}\left[ \mathbf W _{\nu \rho } \mathbf W ^{\nu \rho }\right] , \end{aligned}$$

9b $$\begin{aligned} {\mathcal {L}}_{M,1}&=-g^2 F_{M_1}\text {tr}\left[ (\mathbf D _\mu \mathbf H )^\dagger (\mathbf D ^\rho \mathbf H )\right] \text {tr}\left[ \mathbf W _{\nu \rho } \mathbf W ^{\nu \mu }\right] , \end{aligned}$$

9c $$\begin{aligned} {\mathcal {L}}_{M,2}&=-g^{\prime 2} F_{M_2}\text {tr}\left[ (\mathbf D _\mu \mathbf H )^\dagger (\mathbf D ^\mu \mathbf H )\right] \text {tr} \left[ \mathbf B _{\nu \rho } \mathbf B ^{\nu \rho }\right] , \end{aligned}$$

9d $$\begin{aligned} {\mathcal {L}}_{M,3}&=-g^{\prime 2} F_{M_3}\text {tr}\left[ (\mathbf D _\mu \mathbf H )^\dagger (\mathbf D ^\rho \mathbf H )\right] \text {tr} \left[ \mathbf B _{\nu \rho } \mathbf B ^{\nu \mu }\right] , \end{aligned}$$

9e $$\begin{aligned} {\mathcal {L}}_{M,4}&=-g g^\prime F_{M_4}\text {tr}\left[ (\mathbf D _\mu \mathbf H )^\dagger \mathbf W _{\nu \rho } (\mathbf D ^\mu \mathbf H ) \mathbf B ^{\nu \rho }\right] , \end{aligned}$$

9f $$\begin{aligned} {\mathcal {L}}_{M,5}&=-g g^\prime F_{M_5}\text {tr}\left[ (\mathbf D _\mu \mathbf H )^\dagger \mathbf W _{\nu \rho } (\mathbf D ^\rho \mathbf H ) \mathbf B ^{\nu \mu }\right] , \end{aligned}$$

9g $$\begin{aligned} {\mathcal {L}}_{M,7}&=-g^2 F_{M_7}\text {tr}\left[ (\mathbf D _\mu \mathbf H )^\dagger \mathbf W _{\nu \rho } \mathbf W ^{\nu \mu } (\mathbf D ^\rho \mathbf H ) \right] \; ; \end{aligned}$$

and eight terms which couple gauge-field currents to themselves:

10a $$\begin{aligned} {\mathcal {L}}_{T_0}&=g^4 F_{T_0}\text {tr}\left[ \mathbf W _{\mu \nu } \mathbf W ^{\mu \nu }\right] \text {tr}\left[ \mathbf W _{\alpha \beta } \mathbf W ^{\alpha \beta } \right] , \end{aligned}$$

10b $$\begin{aligned} {\mathcal {L}}_{T_1}&=g^4 F_{T_1}\text {tr}\left[ \mathbf W _{\alpha \nu } \mathbf W ^{\mu \beta }\right] \text {tr}\left[ \mathbf W _{\mu \beta } \mathbf W ^{\alpha \nu }\right] , \end{aligned}$$

10c $$\begin{aligned} {\mathcal {L}}_{T_2}&=g^4 F_{T_2}\text {tr}\left[ \mathbf W _{\alpha \mu } \mathbf W ^{\mu \beta }\right] \text {tr}\left[ \mathbf W _{\beta \nu } \mathbf W ^{\nu \alpha }\right] , \end{aligned}$$

10d $$\begin{aligned} {\mathcal {L}}_{T_5}&=g^2 g^{\prime 2} F_{T_5}\text {tr} \left[ \mathbf W _{\mu \nu } \mathbf W ^{\mu \nu }\right] \text {tr}\left[ \mathbf B _{\alpha \beta } \mathbf B ^{\alpha \beta }\right] , \end{aligned}$$

10e $$\begin{aligned} {\mathcal {L}}_{T_6}&=g^2 g^{\prime 2} F_{T_6}\text {tr} \left[ \mathbf W _{\alpha \nu } \mathbf W ^{\mu \beta }\right] \text {tr}\left[ \mathbf B _{\mu \beta } \mathbf B ^{\alpha \nu }\right] , \end{aligned}$$

10f $$\begin{aligned} {\mathcal {L}}_{T_7}&=g^2 g^{\prime 2} F_{T_7}\text {tr} \left[ \mathbf W _{\alpha \mu } \mathbf W ^{\mu \beta }\right] \text {tr}\left[ \mathbf B _{\beta \nu } \mathbf B ^{\nu \alpha }\right] , \end{aligned}$$

10g $$\begin{aligned} {\mathcal {L}}_{T_8}&=g^{\prime 4} F_{T_8}\text {tr}\left[ \mathbf B _{\mu \nu } \mathbf B ^{\mu \nu }\right] \text {tr}\left[ \mathbf B _{\alpha \beta } \mathbf B ^{\alpha \beta }\right] , \end{aligned}$$

10h $$\begin{aligned} {\mathcal {L}}_{T_9}&=g^{\prime 4} F_{T_9}\text {tr}\left[ \mathbf B _{\alpha \mu } \mathbf B ^{\mu \beta }\right] \text {tr}\left[ \mathbf B _{\beta \nu } \mathbf B ^{\nu \alpha }\right] . \end{aligned}$$

Note that the enumeration of operators is not consecutive. We have adopted the naming convention from the literature [19–21] but eliminated redundant interactions to arrive at a linearly independent set.

We emphasize that this list only describes the model-independent low-energy limit of the true amplitude. The actual measurement of VBS processes is not restricted to the low-energy range and thus cannot be accurately accounted for by the low-energy limit only. The true quasi-elastic scattering amplitudes will resolve the local operators into non-local interactions and thus keep the result in accordance with the applicable unitarity relations at all energies. This is the rationale for introducing simplified models such as (i) and (ii) above.

For the purpose of this study, we adopt a simplification that applies to all considered models: we impose global custodial symmetry on the beyond the SM (BSM) interactions and thus omit all terms that involve the hypercharge boson. In the local operator basis, we are left with two parameters $F_{S_{0/1}}$, three parameters $F_{M_{0/1/7}}$, and three parameters $F_{T_{0/1/2}}$. This choice implies that *W* and *Z* amplitudes are mutually related, and that photon interactions do not carry independent information.

## Properties of the scattering matrix

We apply the phenomenological description of the preceding section to the set of VBS scattering amplitudes, which we then embed in complete LHC processes. The basic processes are all of the $2\rightarrow 2$ quasi-elastic scattering type. In this situation, standard scattering theory applies. We may evaluate partial-wave amplitudes and thus convert the scattering amplitudes to a finite-dimensional matrix. This allows us to diagonalize the scattering matrix and find a unitary projection of each eigenamplitude individually, if the calculated model amplitude does not respect partial-wave unitarity.

This simplification is based on approximations. We ignore external and internal photons. This implies the custodial-*SU*(2) limit, as already discussed in the previous section. It also implies that we ignore the Coulomb pole in charged-*W* scattering. The omission is justified since the forward region is cut out in an experimental analysis, while the Coulomb singularity is not reached for the complete process, due to the spacelike nature of the incoming virtual vector bosons. We also treat the external particles as on-shell, while in the real process at the LHC, the initial vector bosons are actually space-like. In effect, these omissions amount to subleading corrections of relative order $m_W^2/\hat{s}$ and ${q_i^2}/{\hat{s}}$ for the $2\rightarrow 2$ quasi-elastic scattering processes, where $q_i$ is the space-like momentum of an incoming vector boson. We note that for VBS kinematics, values $|q_i^2|\sim m_W^2$ dominate the cross section, but there is a phase-space region where terms proportional to ${q^2}/{\hat{s}}$ become leading. In the current paper, we focus on observables inclusive in $q^2$ where these terms are mostly subleading. We refer to Ref. [22] for a more exhaustive discussion.

In fact, the symmetry structure of our simplified models allows for a choice of basis that renders the scattering matrix diagonal at all energies, up to subleading corrections. Asymptotically, the longitudinal vector boson modes combine with the Higgs mode, while the transverse modes decouple. The external states combine to multiplets of the custodial *SU*(2) symmetry. This property is well known for the SM. If we assume custodial symmetry also for the new interactions, we can use it for expressing all quasi-elastic scattering amplitudes of Higgs and longitudinal vector boson modes in terms of a single scalar master amplitude, which can be used to find partial-wave eigenamplitudes and their unitary projection [7, 23, 24]. Here, we apply the same principle to transverse and mixed scattering amplitudes.

The key observation is that the contact interactions of the SMEFT (8, 9, 10), although they do not provide a satisfactory phenomenological description, already encode the most general dependence of the scattering matrix on external quantum numbers, if we restrict the analysis to quasi-elastic $2\rightarrow 2$ scattering. To describe an arbitrary new-physics spectrum, not just the low-energy limit, we merely have to promote the coefficients $F_i$ to scalar form-factors which can depend on *s*, *t*, *u*. Turning this around, we can formally diagonalize the scattering matrix in terms of those coefficients. Unlike the scattering matrix for longitudinal modes only, this procedure involves the helicities of the external vector bosons $\lambda _i$. Since the procedure is required only for the high-energy range, we neglect the masses of *W*, *Z*, and *H* where applicable.

For the calculation below, we can thus treat the non-SM part of the amplitudes as if they were given by the local dimension-eight operator approximation, keeping in mind that the method works as well for non-constant coefficients. The unitary projection that we obtain assumes the same form, with specific functions for the coefficients, and applying the same projection a second time will not change the asymptotic form of the result anymore.

For the transverse interactions with structures ${\mathcal {L}}_{T_{0/1/2}}$ and for the mixed interactions with the operators ${\mathcal {L}}_{M_{0/1/7}}$ we define the master amplitude

11 $$\begin{aligned}&A(s,t,u;\lambda _1,\lambda _2,\lambda _3,\lambda _4) := \nonumber \\&{\mathcal {A}} (W^+_{\lambda _1} W^-_{\lambda _2} \rightarrow Z_{\lambda _3} Z_{\lambda _4}) \; = \nonumber \\&\quad -2 \, g^4 \, \Bigg (F_{T_0}+\frac{1}{4}F_{T_2}\Bigg ) \delta _{\lambda _1,\lambda _2} \delta _{\lambda _3,\lambda _4}s^2 \nonumber \\&\quad - \, g^4 \, \Bigg (F_{T_1}+\frac{1}{2}F_{T_2}\Bigg ) \nonumber \\&\qquad \quad \cdot \left( \delta _{\lambda _1,-\lambda _3} \delta _{\lambda _2,-\lambda _4}t^2+ \delta _{\lambda _1,-\lambda _4} \delta _{\lambda _2,-\lambda _3}u^2 \right) \nonumber \\&\quad +\frac{1}{2} \, g^4 \, F_{T_2} \, \delta _{\lambda _1,\lambda _2} \delta _{\lambda _3, \lambda _4} \delta _{\lambda _1,-\lambda _3}(t^2+u^2) \nonumber \\&\quad + \frac{1}{16} \, g^2 \, (8 F_{M_0} - 2 F_{M_1} + F_{M_7}) s^2 \nonumber \\&\qquad \quad \cdot \left( \delta _{\lambda _1,\lambda _2} \delta _{\lambda _3,0} \delta _{\lambda _4,0} - \delta _{\lambda _3,\lambda _4} 16\delta _{\lambda _1,0} \delta _{\lambda _2,0} \right) \nonumber \\&\quad + \frac{1}{16} \, g^2 \, (2 F_{M_1} + F_{M_7}) \left( s^2 - t^2 - u^2 \right) \nonumber \\&\qquad \quad \cdot \left( \delta _{\lambda _1,-\lambda _2} \delta _{\lambda _3,0} \delta _{\lambda _4,0} - \delta _{\lambda _3,-\lambda _4} \delta _{\lambda _1,0} \delta _{\lambda _2,0} \right) \nonumber \\&\quad + \frac{1}{16} \, g^2 \, F_{M_7} \cdot \nonumber \\&\qquad \quad \biggl [ \left( \delta _{\lambda _1,-\lambda _3} \delta _{\lambda _2,0} \delta _{\lambda _4,0} -\delta _{\lambda _2,-\lambda _4} \delta _{\lambda _1,0} \delta _{\lambda _3,0} \right) \left( s^2 - u^2 \right) \nonumber \\&\qquad \quad + \left( \delta _{\lambda _2,-\lambda _3} \delta _{\lambda _1,0} \delta _{\lambda _4,0} -\delta _{\lambda _1,-\lambda _4} \delta _{\lambda _2,0} \delta _{\lambda _3,0} \right) \left( s^2 - t^2 \right) \biggr ]. \end{aligned}$$

The decomposition of the scattering amplitudes into isospin eigenamplitudes is identical for mixed and transverse operators and given by

12a $$\begin{aligned} {\mathcal {A}}(W^+_{\lambda _1} W^+_{\lambda _2} \rightarrow W^+_{\lambda _3} W^+_{\lambda _4})&=\phantom {\frac{1}{3}}{\mathcal {A}}_2 (s,t,u; {\varvec{\lambda }}) \end{aligned}$$

12b $$\begin{aligned} {\mathcal {A}}(W^+_{\lambda _1} W^-_{\lambda _2} \rightarrow W^+_{\lambda _3} W^-_{\lambda _4})&=\frac{1}{3}{\mathcal {A}}_0 (s,t,u;{\varvec{\lambda }})+ \frac{1}{2}{\mathcal {A}}_1 (s,t,u;{\varvec{\lambda }}) \nonumber \\&\qquad \;+\frac{1}{6} {\mathcal {A}}_2 (s,t,u;{\varvec{\lambda }}) \end{aligned}$$

12c $$\begin{aligned} {\mathcal {A}}(W^+_{\lambda _1} W^-_{\lambda _2} \rightarrow Z_{\lambda _3} Z_{\lambda _4})&=\frac{1}{3}{\mathcal {A}}_0 (s,t,u;{\varvec{\lambda }})- \frac{1}{3}{\mathcal {A}}_2 (s,t,u;{\varvec{\lambda }}) \end{aligned}$$

12d $$\begin{aligned} {\mathcal {A}}(W^+_{\lambda _1} Z_{\lambda _2} \rightarrow W^+_{\lambda _3} Z_{\lambda _4})&=\frac{1}{2}{\mathcal {A}}_1 (s,t,u;{\varvec{\lambda }})+ \frac{1}{2}{\mathcal {A}}_2 (s,t,u;{\varvec{\lambda }}) \end{aligned}$$

12e $$\begin{aligned} {\mathcal {A}}(Z_{\lambda _1} Z_{\lambda _2} \rightarrow Z_{\lambda _3} Z_{\lambda _4})&=\frac{1}{3}{\mathcal {A}}_0 (s,t,u;{\varvec{\lambda }})+ \frac{2}{3}{\mathcal {A}}_2 (s,t,u;{\varvec{\lambda }}) \; . \end{aligned}$$

Here, ${{\varvec{\lambda }}} =(\lambda _1,\lambda _2,\lambda _3,\lambda _4)$ is a multi-index for the four different helicities of the weak vector bosons. Using this, the isospin eigenamplitudes are given by

13a $$\begin{aligned} {\mathcal {A}}_0(s,t,u;{\varvec{\lambda }}) =&\quad \; 3A(s,t,u;\lambda _1,\lambda _2,\lambda _3,\lambda _4) \nonumber \\&+A(t,s,u;-\lambda _4,\lambda _2,\lambda _3,-\lambda _1) \nonumber \\&+A(u,t,s;\lambda _1,-\lambda _4,\lambda _3,-\lambda _2) \end{aligned}$$

13b $$\begin{aligned} {\mathcal {A}}_1(s,t,u;{\varvec{\lambda }}) =&\quad \; A(t,s,u;-\lambda _4,\lambda _2,\lambda _3,-\lambda _1) \nonumber \\&-A(u,t,s;\lambda _1,-\lambda _4,\lambda _3,-\lambda _2) \end{aligned}$$

13c $$\begin{aligned} {\mathcal {A}}_2(s,t,u;{\varvec{\lambda }}) =&\quad \; A(t,s,u;-\lambda _4,\lambda _2,\lambda _3,-\lambda _1) \nonumber \\&+A(u,t,s;\lambda _1,-\lambda _4,\lambda _3,-\lambda _2). \end{aligned}$$

The next step is the decomposition into isospin-spin eigenamplitudes which is done by the expansion of the isospin eigenamplitudes (12) into the Wigner D-functions [25] $d^J_{\lambda ,\lambda ^\prime }(\theta )$ with $\lambda =\lambda _1-\lambda _2$ and $\lambda ^\prime =\lambda _3-\lambda _4$.

14 $$\begin{aligned} {\mathcal {A}}_{IJ}(s;{\varvec{\lambda }})=\int _{-s}^0\frac{dt}{s} A_I(s,t,u;{\varvec{\lambda }}) \cdot d^J_{\lambda ,\lambda ^\prime }\left[ \arccos \left( 1+2\frac{t}{s}\right) \right] \end{aligned}$$

Table 1 (transverse operators) and Table 2 (mixed operators) list the complete set of master amplitudes with their dependence on the operator coefficients, i.e., the asymptotically leading behavior. These helicity-dependent eigenamplitudes can in principle be used for determining unitary projections as form factors that multiply modified Feynman rules for the boson fields.

**Table 1:** Coefficients of the isospin-spin amplitudes calculated with Eq. (14) for the mixed operators ${\mathcal {L}}_{M_i}$ depending on the helicity of the incoming and outgoing particles. The isospin spin amplitudes are given by $A_{ij}(s; {\varvec{\lambda }})=(c_0 F_{M_0} + c_1 F_{M_1} + c_2 F_{M_7})g^2 s^2$

i	j												
	0			1			2			${\varvec{\lambda }}$			
0	$\frac{3}{2}$	$-\frac{3}{8}$	$\frac{3}{16}$	0	0	0	0	0	0	$+$	$+$	0	0
	0	0	0	0	0	0	0	$\frac{1}{20}\sqrt{\frac{3}{2}}$	$\frac{1}{40}\sqrt{\frac{3}{2}}$	$+$	−	0	0
	0	0	0	$-\frac{1}{8}$	$\frac{1}{32}$	$\frac{7}{192}$	$\frac{1}{40}$	$-\frac{1}{160}$	$\frac{3}{320}$	$+$	0	−	0
	0	0	0	$\frac{1}{8}$	$-\frac{1}{32}$	$-\frac{7}{192}$	$\frac{1}{40}$	$-\frac{1}{160}$	$\frac{3}{320}$	$+$	0	0	−
	0	0	0	0	$\frac{1}{12}$	$\frac{1}{24}$	0	0	0	$+$	0	$+$	0
	0	0	0	0	$-\frac{1}{12}$	$-\frac{1}{24}$	0	0	0	$+$	0	0	$+$
1	0	0	0	0	0	$\frac{1}{24}$	0	0	0	$+$	$+$	0	0
	0	0	0	0	0	0	0	0	0	$+$	−	0	0
	0	0	0	$-\frac{1}{8}$	$\frac{1}{32}$	$\frac{1}{96}$	$\frac{1}{40}$	$-\frac{1}{160}$	$\frac{1}{160}$	$+$	0	−	0
	0	0	0	$-\frac{1}{8}$	$\frac{1}{32}$	$\frac{1}{96}$	$-\frac{1}{40}$	$\frac{1}{160}$	$-\frac{1}{160}$	$+$	0	0	−
	0	0	0	0	$\frac{1}{12}$	$\frac{1}{24}$	0	0	0	$+$	0	$+$	0
	0	0	0	0	$\frac{1}{12}$	$\frac{1}{24}$	0	0	0	$+$	0	0	$+$
2	0	0	0	0	0	0	0	0	0	$+$	$+$	0	0
	0	0	0	0	0	0	0	0	0	$+$	−	0	0
	0	0	0	$-\frac{1}{8}$	$\frac{1}{32}$	$-\frac{1}{24}$	$\frac{1}{40}$	$-\frac{1}{160}$	0	$+$	0	−	0
	0	0	0	$\frac{1}{8}$	$-\frac{1}{32}$	$\frac{1}{24}$	$\frac{1}{40}$	$-\frac{1}{160}$	0	$+$	0	0	−
	0	0	0	0	$\frac{1}{12}$	$\frac{1}{24}$	0	0	0	$+$	0	$+$	0
	0	0	0	0	$-\frac{1}{12}$	$-\frac{1}{24}$	0	0	0	$+$	0	0	$+$
	$c_0$	$c_1$	$c_2$	$c_0$	$c_1$	$c_2$	$c_0$	$c_1$	$c_2$

**Table 2:** Coefficients of the isospin-spin amplitudes calculated with Eq. (14) for the transversal operators ${\mathcal {L}}_{T_i}$ depending on the helicity of the incoming and outgoing particles. The isospin-spin amplitudes are given by $A_{ij}(s; {\varvec{\lambda }})=(c_0 F_{T_0} + c_1 F_{T_1} + c_2 F_{T_2})g^4 s^2$

i	j												
	0			1			2			${\varvec{\lambda }}$			
0	$-$ 6	$-$ 2	$-\frac{5}{2}$	0	0	0	0	0	0	$+$	$+$	$+$	$+$
	0	0	0	0	0	0	$-\frac{2}{5}$	$-\frac{4}{5}$	$-\frac{1}{2}$	$+$	−	$+$	−
	0	0	0	0	0	0	$-\frac{2}{5}$	$-\frac{4}{5}$	$-\frac{1}{2}$	$+$	−	−	$+$
	$-\frac{22}{3}$	$-\frac{14}{3}$	$-\frac{11}{6}$	0	0	0	$-\frac{2}{15}$	$-\frac{4}{15}$	$-\frac{1}{30}$	$+$	$+$	−	−
1	0	0	0	0	0	0	0	0	0	$+$	$+$	$+$	$+$
	0	0	0	0	0	0	$\frac{2}{5}$	$-\frac{1}{5}$	0	$+$	−	$+$	−
	0	0	0	0	0	0	$-\frac{2}{5}$	$\frac{1}{5}$	0	$+$	−	−	$+$
	0	0	0	$\frac{2}{3}$	$-\frac{1}{3}$	$\frac{1}{6}$	0	0	0	$+$	$+$	−	−
2	0	$-$ 2	$-$ 1	0	0	0	0	0	0	$+$	$+$	$+$	$+$
	0	0	0	0	0	0	$-\frac{2}{5}$	$-\frac{1}{5}$	$-\frac{1}{5}$	$+$	−	$+$	−
	0	0	0	0	0	0	$-\frac{2}{5}$	$-\frac{1}{5}$	$-\frac{1}{5}$	$+$	−	−	$+$
	$-\frac{4}{3}$	$-\frac{8}{3}$	$-\frac{1}{3}$	0	0	0	$-\frac{2}{15}$	$-\frac{1}{15}$	$-\frac{1}{30}$	$+$	$+$	−	−
	$c_0$	$c_1$	$c_2$	$c_0$	$c_1$	$c_2$	$c_0$	$c_1$	$c_2$				

For the purpose of constructing a minimal unitary projection, it is sufficient to determine a set of master amplitudes which capture the leading term proportional to $s^2$ for each spin-isospin channel, uniformly for all individual helicity combinations. The implied over-compensation of some helicity channels that are subleading at high energy, is within the scheme dependence that is inherent in the unitary projection. We find the following simplified, helicity-independent expressions:

15a $$\begin{aligned} {\mathcal {A}}_{00}(s) =&- \frac{3}{2} g^4 \left[ 4 F_{T_0} - 2 F_{T_1} + F_{T_2}\right] s^2 \nonumber \\&+ \frac{3}{16} g^2 \left[ 8 F_{M_0} + 2 F_{M_1} + F_{M_7} \right] s^2 \end{aligned}$$

15b $$\begin{aligned} {\mathcal {A}}_{01}(s) =&- \frac{1}{32} g^2 \left[ 4 F_{M_0} + F_{M_1} - 3 F_{M_7} \right] s^2 \end{aligned}$$

15c $$\begin{aligned} {\mathcal {A}}_{02}(s) =&- \frac{1}{10} g^4 \left[ 4 F_{T_0} - 2 F_{T_1} + F_{T_2}\right] s^2 \nonumber \\&+ \frac{1}{160} g^2 \left[ 4 F_{M_0} + F_{M_1} + F_{M_7} \right] s^2 \end{aligned}$$

15d $$\begin{aligned} {\mathcal {A}}_{10}(s) =&0 \end{aligned}$$

15e $$\begin{aligned} {\mathcal {A}}_{11}(s) =&- \frac{1}{6} g^4 F_{T_2} s^2 \nonumber \\&- \frac{1}{32} g^2 \left[ 4 F_{M_0} + F_{M_1} - 3 F_{M_7}\right] s^2 \end{aligned}$$

15f $$\begin{aligned} {\mathcal {A}}_{12}(s) =&\frac{1}{5} g^4 \left[ - 2 F_{T_0} + F_{T_1}\right] s^2 \nonumber \\&+ \frac{1}{160} g^2 \left[ 4 F_{M_0} + F_{M_1} + F_{M_7} \right] s^2 \end{aligned}$$

15g $$\begin{aligned} {\mathcal {A}}_{20}(s) =&0 \end{aligned}$$

15h $$\begin{aligned} {\mathcal {A}}_{21}(s) =&- \frac{1}{32} g^2 \left[ 4 F_{M_0} + F_{M_1} - 3 F_{M_7} \right] s^2 \end{aligned}$$

15i $$\begin{aligned} {\mathcal {A}}_{22}(s) =&- \frac{1}{10} g^4 \left[ 4 F_{T_0} - 2 F_{T_1} + F_{T_2}\right] s^2 \nonumber \\&+ \frac{1}{160} g^2 \left[ 4 F_{M_0} + F_{M_1} + F_{M_7} \right] s^2. \end{aligned}$$

In fact, comparing polarized $2\rightarrow 2$ on-shell processes we have verified that the numerical discrepancy between a helicity-dependent treatment and the simplified version is less than a percent for the parameter ranges considered, negligible in comparison to the scheme dependence of the unitary projection itself.

We now turn to the unitary projection of the scattering amplitudes, applicable to the high-energy range where the leading behavior in the presence of nonzero operator coefficients is given by (15). We follow the T-matrix projection scheme introduced in Ref. [7] and apply it to the simplified helicity-independent eigenamplitudes, for the case where only one type of new interactions (mixed or transversal) is active at a time. The simplifications combined allow us to evaluate the projection and thus the compensating terms in closed form. (If coefficients are non-zero for both classes simultaneously or a detailed separation of helicities is intended, we have to resort to numerical evaluation of the T-matrix projection. This is beyond the scope of the present work.)

The unitary projection of a spin-isospin eigenamplitude ${{\mathcal {A}}}_{IJ}$ is given by the expression

16 $$\begin{aligned} {\hat{{\mathcal {A}}}}_{IJ} = \frac{1}{ \mathrm {Re} {\frac{1}{{\mathcal {A}}_{IJ}}}- \frac{\mathrm {i}}{32 \pi }}. \end{aligned}$$

This projection may be recast as an *s*-dependent correction counterterm for each eigenamplitude,

17 $$\begin{aligned} \varDelta {\mathcal {A}}_{IJ} = {\hat{{\mathcal {A}}}}_{IJ} - {\mathcal {A}}_{IJ}. \end{aligned}$$

The limit $A_{IJ}\rightarrow \infty$ lets us recover the universal unitarity bound for each eigenamplitude,

18 $$\begin{aligned} |{\hat{{\mathcal {A}}}}_{IJ}| \le 32 \pi . \end{aligned}$$

In particular, the truncated SMEFT expansion, i.e. constant coefficients in the eigenamplitude above, yields a limit $\lim _{s\rightarrow \infty }{\mathcal {A}}_{IJ}\rightarrow \infty$ for all partial waves with nonzero coefficients. The T-matrix projection then asymptotically saturates unitarity. A model with a pole in the amplitude at some value $s=M^2$, projected according to this prescription, saturates the unitarity limit at this point and follows a Breit-Wigner shape for the energy dependence in the vicinity of the pole. The actual pole of the amplitude gets shifted away from the real axis.

## (Strongly coupled) continuum model

As mentioned in Sect. 2, the simplified models that we actually consider are (i) continuum models which smoothly interpolate between high-energy unitarity saturation and the low-energy SMEFT, and (ii) resonance models where distinct features arise in the spectrum. Including also the unmodified SM in the discussion, these models cover the whole range of possible interaction strengths that future VBS measurements may observe, and thus yield a fairly robust projection for the sensitivity of a collider experiment. With the exception of the unmodified SM, neither of these models is UV complete, and the actual results should behave differently in the asymptotic regime. For instance, new inelastic channels may appear as final states. However, as long as the initial assumptions about unitarity, gauge invariance and minimal flavor violation hold true, we should not expect event rates in this sector which exceed the strongly-interacting continuum scenario that we consider here.

For our numerical studies of the continuum scenario, we have adopted the amplitudes with the local operators of Sect. 2 added to the SM Feynman rules and converted this to a unitary model according to (16) after diagonalization. This has been re-expressed in terms of form-factor modified Feynman rules along the lines of Refs. [7, 23] and implemented in the Monte-Carlo event generator WHIZARD [26].^3^ Using Feynman rules and a straightforward on-shell projection of the boson momenta, the interactions can be evaluated off-shell in the context of an automatic amplitude evaluation, and thus enter the standard WHIZARD framework that ultimately yields simulated event samples. There are some subtleties hidden in the on-shell projection; this is discussed in detail, along with some refinement of the method, in Ref. [22]. All ingredients for the simulation of the transversal and mixed operators (as well as for the longitudinal operators discussed in [7]) as well as the resonances for the simplified models to be detailed in Sec. 5 can be found in the model SSC2 within the official WHIZARD release from version 2.6.2 onwards.

**Fig. 1:**
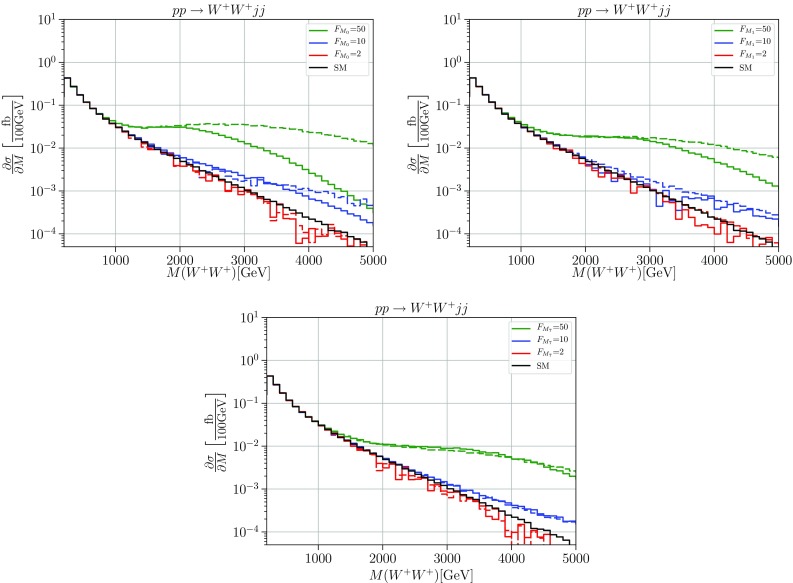
Cross sections differential in the diboson invariant mass for the process $pp \rightarrow W^+W^+jj$. The solid black line shows the Standard Model differential cross section, the green, blue and red lines the cross sections with anomalous couplings $F_{M_i}=50\,\ \text {TeV}^{-4}, F_{M_i}=10\,\text {TeV}^{-4}$ and $F_{M_i}=2\,\text {TeV}^{-4}$ for i = 0 (upper left panel), i = 1 (upper right panel), and i = 7 (lower panel), respectively. Solid: unitarized; dashed: naive result. Cuts: $M_{jj}>500\,\ \text {GeV}$, $\varDelta \eta _{jj}>2.4$, $|\eta _j|<4.5$, $p_T^j>20\,\ \text {GeV}$

**Fig. 2:**
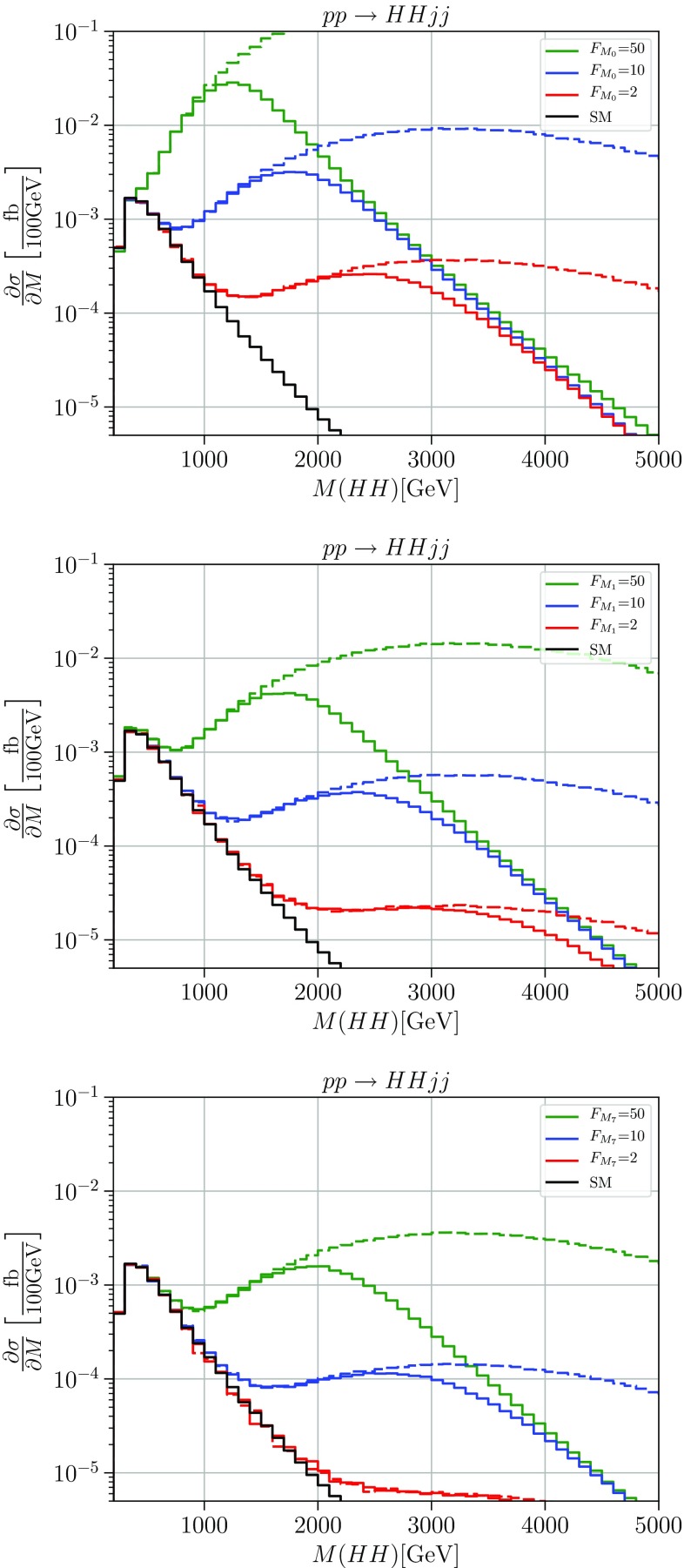
Cross sections differential in the diboson invariant mass for the process $pp \rightarrow HHjj$. The solid black line shows the Standard Model differential cross section, the green, blue and red lines the cross sections with anomalous couplings $F_{M_i}=50\,\ \text {TeV}^{-4}, F_{M_i}=10\,\text {TeV}^{-4}$ and $F_{M_i}=2\,\text {TeV}^{-4}$ for i = 0 (upper panel), i = 1 (middle panel), and i = 7 (right panel), respectively. Solid: unitarized; dashed: naive result. Cuts: $M_{jj}>500\,\ \text {GeV}$, $\varDelta \eta _{jj}>2.4$, $|\eta _j|<4.5$, $p_T^j>20\,\ \text {GeV}$

The processes $pp\rightarrow jjW^+W^+$ and $pp\rightarrow jjHH$ have received special attention. The former process exhibits a characteristic signature of like-sign dileptons and has the largest signal-to-branching ratio of all VBS processes at the LHC, while the latter is difficult to isolate but carries a dependence on the triple-Higgs coupling which is among the most elusive SM parameters. In fact, an anomalous triple-Higgs coupling can be attributed to a gauge-invariant dimension-six operator, while in this work we are considering dimension-eight contributions. Clearly, an unambiguous determination of a dimension-six parameter in a systematic low-energy expansion is only possible if the next higher order is under control.

In Figs. 1 and  2 we show results for the process $pp \rightarrow W^+W^+ jj$ and for $pp \rightarrow HHjj$ within the continuum simplified model with nonzero coefficients for the longitudinal-transverse mixed operators with parameters $F_{M_{0/1/7}}$, respectively. We choose three distinct values, $F=2,10,50\;\text {TeV}^{-4}$, with one nonzero coefficient at a time. The solid lines show the distribution in the invariant mass of the $W^+W^+/HH$ pair, which coincides with the effective energy $\sqrt{\hat{s}}$ for the basic VBS process. We note that in the presence of background and finite jet-energy resolution, this distribution is not actually measurable for $W^+W^+$. Rather, *W*-boson decay leptons are detected. However, the plots describe most clearly the expected physics, which will only be diluted in actual observables.

For reference, we also display the results which would be obtained if the naive dimension-eight SMEFT amplitude, without T-matrix correction, were used for the calculation (dashed). Clearly, such a calculation overestimates the achievable event yield by a huge amount and suggests a sensitivity to the model parameters which is unphysical.

We observe that regardless of parameters, the solid curves approach an asymptotic differential cross section which for the $W^+W^+$ final state is enhanced by about an order of magnitude over the SM prediction. In the case of the *HH* final state, the enhancement amounts to more than two orders of magnitude. These asymptotic limits correspond to a maximally strong interaction, saturation of the unitarity limit within the quasi-elastic channel. The residual parameter dependence is confined to a certain transition region. Beyond this region, from the saturated quasi-elastic amplitudes we can read off the maximally allowed event number for the given spin-isospin channel.

**Fig. 3:**
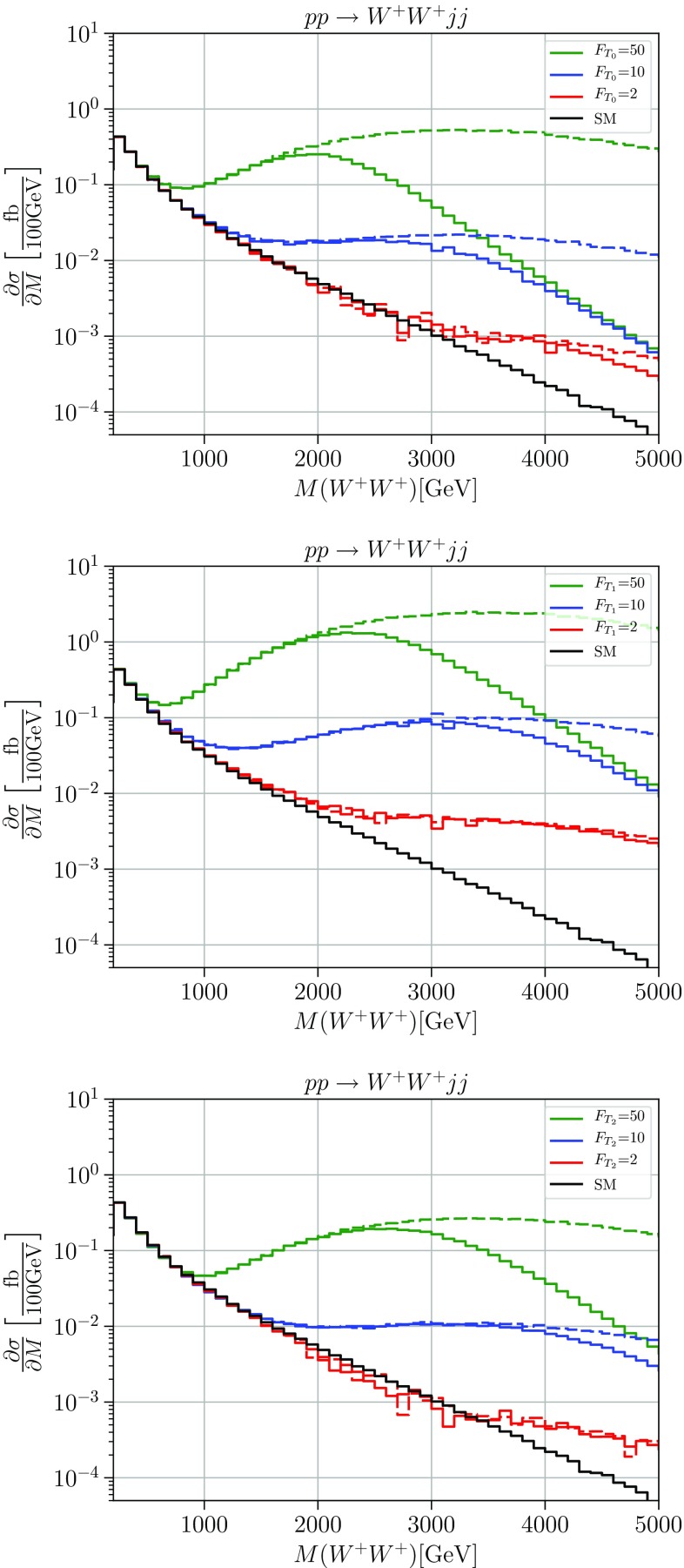
Cross sections differential in the diboson invariant mass for the process $pp \rightarrow W^+W^+jj$. The solid black line shows the SM differential cross section, the green, blue and red lines the cross sections with anomalous couplings $F_{T_i}=50\,\ \text {TeV}^{-4}, F_{T_i}=10\,\text {TeV}^{-4}$ and $F_{T_i}=2\,\text {TeV}^{-4}$ for i = 0 (upper panel), i = 1 (middle panel), and i = 2 (lower panel), respectively. Solid: unitarized; dashed: naive result. Cuts: $M_{jj}>500\,\ \text {GeV}$, $\varDelta \eta _{jj}>2.4$, $|\eta _j|<4.5$, $p_T^j>20\,\ \text {GeV}$

In Fig. 3 we plot results for the purely transverse interactions with parameters $F_{T_{0/1/2}}$. Again, the studied process is $pp \rightarrow W^+W^+ jj$. The *HH* channel is not affected by these interactions, because the purely transverse operators do not contribute to any anomalous coupling involving a Higgs. We choose three distinct values, $F_T=2,10,50\;\text {TeV}^{-4}$, with one nonzero coefficient at a time.

In these scenarios, the asymptotic enhancement of the continuum model over the SM approaches two orders of magnitude. We may read this observation as an indication for much larger freedom for new-physics effects in purely transverse vector-boson interactions, compared to mixed and purely longitudinal interactions. This fact should be accounted for in data analysis. Nevertheless, also in this class of models, the naive SMEFT result represented by the dashed lines overestimates the possible event rates by a large factor.

We emphasize that the above plots, which only indicate the variation with respect to one of the model parameters, should not be taken individually as realistic predictions, even if accepting the basic assumptions regarding a strongly interacting continuum in the electroweak sector. They sweep a range of predictions, within the given model class. In reality, we expect more than one coefficient to be present, so a global fit would be required to determine the correct parameter dependence and the sensitivity of a collider experiment. On the other hand, we can already conclude that due to the failure of the naive SMEFT, there is no meaningful description of these processes that can be viewed as model-independent.

“Failure” of the SMEFT here is not to be understood such that there is no quantum field theoretically valid (truncated) low-energy expansion of a UV-complete model. It rather expresses the practical problem of the tension between a size of operator coefficients experimentally detectable at the LHC and the limitations from the unitarity bounds. Numerical results of non-SM interactions of longitudinal scattering have clearly shown that for the level of deviations that can be detected by the LHC experiments, the unitarity limits are always violated in the high-energy range, if a naive SMEFT calculation is attempted. The study presented in [22] substantiates that also transversal and mixed dimension-8 operators will start violating pertubative unitarity at diboson invariant masses between 1 and 2 TeV. Using only the SMEFT leads to overconstrained limits of the dimension-8 operators by a factor of two to three. This was also exemplified by the Warsaw group in Ref. [8] studying the parameter space of low-energy EFTs of UV-complete models in the plane of the Wilson coefficients and the scale of new physics: in the upper right corner of large coefficients and large scales, unitarity destroys the viability. For small coefficients and not too low scales, LHC cannot detect any deviations, while for large coefficients and too low scales, the EFT expansion breaks down, leaving only a triangular region that is both theoretically allowed and experimentally accessible. Reference [8] has studied UV models where this region is actually vanishing. An analysis that compares actual data to a prediction, apart from the SM result, must choose among the conceivable (simplified) models for a comparison, of which we can only show a set of examples.

If several anomalous couplings are present in a model, it is essential to increase the number of independent observables that enter a global fit of all parameters. At the LHC, there is a number of di-boson final states that can be produced in VBS. In Appendix B, Figs. 6, 7, 8, we present results for the additional VBS final states $W^+W^-$, $W^+Z$, and *ZZ* which are not as easily accessible or have smaller leptonic rates compared to $W^+W^+$ but should be considered in this context, particularly as there are already results from the LHC experiments for the latter two. For those results, we choose a value of $2\;\text {TeV}^{-4}$ for each of the parameters $F_i$, with only one parameter nonzero at a time.

## Simplified resonance models

In this section, we consider simplified models where an anomalous local interaction resolves into a resonance which saturates a partial-wave spin-isospin amplitude. A resonance saturates an elastic channel for finite energy and exhibits a falloff of the amplitude beyond the peak, before strong interactions may set in again at higher energy. This is observed, e.g., for some isolated bound states that precede a strongly interacting continuum in QCD. In Ref. [9], we described this class of models in the context of VBS and studied couplings of the resonance to longitudinal gauge bosons via the scalar current $J_H^{(4)}$ (5). In this work, we extend the allowed coupling to transversal bosons. As an example, we take a single scalar with a coupling to the current $J^{(4)}_W$ (6).

There are various models of a non-minimal Higgs sector which effectively lead to a phenomenology of this type. In general, we expect couplings of the resonance both to longitudinal and transverse vector-boson modes. BSM models which allow a direct coupling of a new physics particle only to the transverse mode of electroweak gauge bosons are often very constrained by data [40]. Only a few extra-dimensional models [41, 42] including a directly and strongly coupled spin-2 resonance, for example a KK-graviton, are not as hampered by experimental data. Other BSM models introduce the coupling of transverse vector bosons to a new physics particle not directly, but due to loop contributions. In Randall-Sundrum [43] or ADD [44] models this could also be achieved through a top loop [45].

Models with extra scalar resonances typically introduce additional new heavy particles. For instance, in composite Higgs models the coupling to the transversal gauge sector can be mediated by technipions [46] or by heavy fermions [47, 48]. If the mass scale of such extra heavy particles is beyond the experimental reach of LHC, the loop contributions are small and can be parametrized within an EFT. Effective couplings of a resonance involve both longitudinal and transversal vector bosons. In recent diphoton studies, this EFT framework was also used to estimate the effect of a possible diphoton resonance [40, 49–51]. General vector resonances in the explicit channel of *WZ* scattering have been studied in [52]. Another class of models with heavy resonances are Little Higgs models [53, 54]. For these models, the coefficients of the SMEFT as the low-energy expansion have been calculated, e.g., in Ref. [55].

In the present paper, we do not refer to a specific scenario. We construct a simplified model with transverse couplings of a generic heavy resonance $\sigma$. The effective Lagrangian takes the following form,

19a $$\begin{aligned} \mathcal {L}_{\sigma }&= - \frac{1}{2} \sigma (m_\sigma ^2 - \partial ^2) \sigma + \sigma (J_{\sigma \parallel }+J_{\sigma \perp }) \end{aligned}$$

19b $$\begin{aligned} J_{\sigma \parallel }&=F_{\sigma H}{\text {tr}}\left[ (\mathbf {D}_\mu \mathbf {H})^\dagger (\mathbf {D}^\mu \mathbf {H})\right] \end{aligned}$$

19c $$\begin{aligned} J_{\sigma \perp }&= g^2 F_{W\sigma }\sigma {\text {tr}}\left[ \mathbf {W}_{\mu \nu }\mathbf {W}^{\mu \nu }\right] + {g^{\prime }}^2F_{B\sigma }\sigma {\text {tr}}\left[ \mathbf {B}_{\mu \nu }\mathbf {B}^{\mu \nu }\right] \end{aligned}$$

with three independent coupling parameters.

In the low-energy limit, the scalar resonance can be integrated out, and we obtain the SMEFT Lagrangian with the following nonzero coefficients of the dimension-8 operators at leading order:

20a $$\begin{aligned} F_{S_0}&= \phantom {-} {F_{\sigma H}^2}/{2 m_\sigma ^2} \end{aligned}$$

20b $$\begin{aligned} F_{M_0}&= -{F_{\sigma H}F_{\sigma W}}/{m_\sigma ^2} \end{aligned}$$

20c $$\begin{aligned} F_{M_2}&= -{F_{\sigma H}F_{\sigma B}}/{m_\sigma ^2} \end{aligned}$$

20d $$\begin{aligned} F_{T_0}&=\phantom {-} {F_{\sigma W}^2}/{2m_\sigma ^2} \end{aligned}$$

20e $$\begin{aligned} F_{T_5}&=\phantom {-} {F_{\sigma W}F_{\sigma B}}/{m_\sigma ^2} \end{aligned}$$

20f $$\begin{aligned} F_{T_8}&= \phantom {-} {F_{\sigma B}^2}/{2m_\sigma ^2}. \end{aligned}$$

To set the relation between the coupling constant to the electroweak currents and the resonance mass, we also compute the width of the scalar resonance:

21 $$\begin{aligned} \varGamma (m_\sigma ) =&\; \int d\varOmega \frac{|\mathbf {p}|}{32 \pi ^2 m_\sigma ^2} \left( |\mathcal {M}_{\sigma \rightarrow W^+W^-}|^2 + \frac{1}{2}|\mathcal {M}_{\sigma \rightarrow ZZ}|^2 \right. \nonumber \\&\left. \; + \frac{1}{2}|\mathcal {M}_{\sigma \rightarrow HH}|^2 + |\mathcal {M}_{\sigma \rightarrow Z\gamma }|^2 + \frac{1}{2}|\mathcal {M}_{\sigma \rightarrow \gamma \gamma }|^2 \right) , \end{aligned}$$

with $|\mathbf {p}|=m_\sigma /2$. Here, we neglect the masses of the electroweak gauge bosons in the kinematics of the phase space vectors.

The model with only $F_{\sigma H}$ nonzero has been covered in Ref. [9]. For this paper, we set $F_{\sigma H}=F_{\sigma B}=0$ and keep only $F_{\sigma W}$. The resonance width becomes

22 $$\begin{aligned} \varGamma _W(m_\sigma )&= \;\frac{3m_\sigma ^3}{16 \pi }g^4F_{\sigma W}^2 \left( 1+ {\mathcal {O}}(1/m^2_\sigma ) \right) . \end{aligned}$$

The low-energy limit contains only the operator ${\mathcal {L}}_{T_{0}}$. We can thus easily compare distributions with a resonance to the anologous distributions with a continuum, where both models reduce to the same low-energy limit. While the low-energy approximation has a single parameter, the dimension-eight operator coefficient $F_{T_0}$, the resonance model has two free parameters, the resonance mass and the resonance coupling, or alternatively the width.

**Fig. 4:**
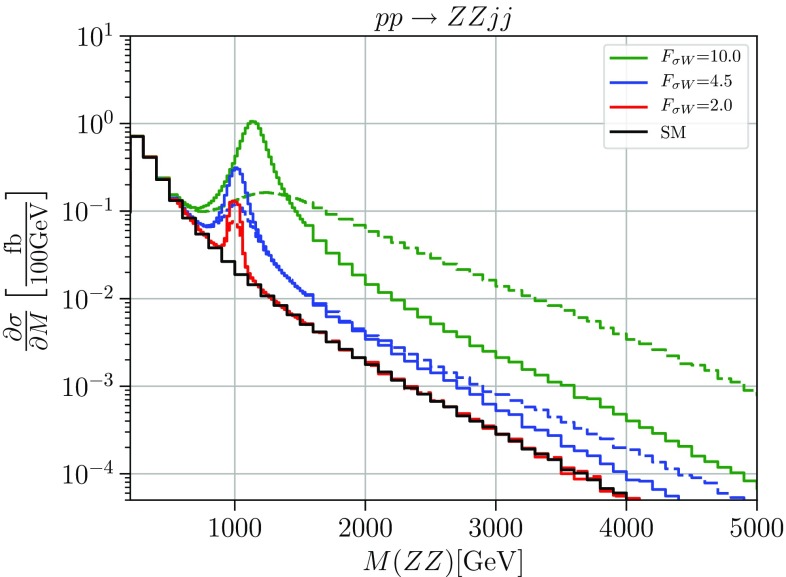
Cross sections differential in the diboson invariant mass for the process $pp \rightarrow ZZjj$. The solid black line shows the Standard Model differential cross section, the green, blue and red lines the cross sections with a scalar resonance with mass $m_\sigma = 1\,\text {TeV}$ and coupling of $F_{W \sigma }=10.0\,\ \text {TeV}^{-1}, F_{W \sigma }=4.5\,\text {TeV}^{-1}$ and $F_{W \sigma }=2.0\,\text {TeV}^{-1}$, respectively. Solid: unitarized; dashed: naive result. Cuts: $M_{jj}>500\,\ \text {GeV}$, $\varDelta \eta _{jj}>2.4$, $|\eta _j|<4.5$, $p_T^j>20\,\ \text {GeV}$

We have implemented the resonance model in the Monte-Carlo generator WHIZARD [26], using the same unitarity projection algorithm as for the continuum models. In Fig. 4, we show the invariant-mass distribution of the *ZZ* final state for a scalar resonance with mass $m_\sigma = 1 \,\mathrm {TeV}$ and different couplings $F_{\sigma W}=10.0,4.5,2.0 \, \text {TeV}^{-1}$. These values correspond to the anomalous quartic coupling $F_{T_0}=50,10,2 \, \text {TeV}^{-4}$ if the scalar resonance is integrated out. The dashed lines show the naive result of implementing the scalar resonance as an extra particle with its width given by the formula (22). The solid lines show the unitary projection for each coupling value, respectively.

This plot illustrates two properties of resonance models. First of all, we observe that the unitary projection has two effects: on the resonance, the peak becomes narrower and more pronounced. This is the result of subleading terms in the width formula, which we did not include in the naive result but which are accounted for by the unitary projection. Asymptotically, the amplitude is suppressed by the projection. This is the result of saturating partial waves by $s^2$ terms which originate from the derivative coupling.

Since a derivative coupling is a typical feature of strong interactions where couplings involve form factors, and a necessary property of resonances with higher spin, the asymptotic effect of unitarity saturation is essential for a complete description. The T-matrix projection is a method for implementing unitarity in the model for the whole kinematical range.

**Fig. 5:**
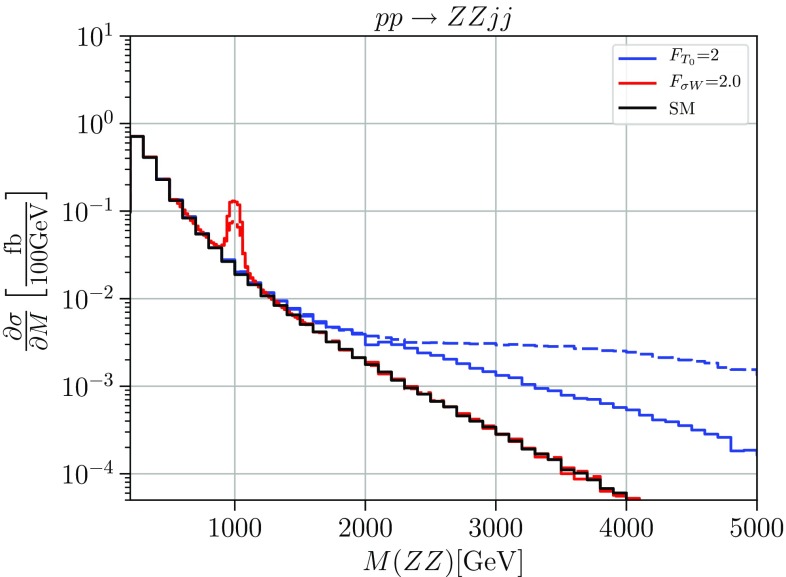
Cross sections differential in the diboson invariant mass for the process $pp \rightarrow ZZjj$. The solid black line shows the Standard Model, the blue lines show an anomalous coupling $F_{T_i}=2\,\text {TeV}^{-4}$ and the red lines show a scalar resonance with mass $m_\sigma = 1\,\text {TeV}$ and coupling of $F_{\sigma W}=2.0\,~\text {TeV}^{-2}$. Solid: unitarized; dashed: naive result. Cuts: $M_{jj}>500\,\ \text {GeV}$, $\varDelta \eta _{jj}>2.4$, $|\eta _j|<4.5$, $p_T^j>20\,\ \text {GeV}$

In Fig. 5, we compare the simplified model with a scalar resonance with mass $m_\sigma = 1 \,\mathrm {TeV}$ and coupling $F_{\sigma W}=2.0 \, \text {TeV}^{-1}$ (red) to the corresponding EFT result with the matching anomalous quartic coupling $F_{T}=2 \,\text {TeV}^{-4}$ (blue), with and without unitary projection (solid vs. dashed). This is a rather small coupling, and the resonance behaves almost like an elementary particle. The peak is not approximated at all by the EFT operator description. We may argue that for such a type of model, the EFT is useful only in the case of strong couplings and broad resonances. We also see that the high-energy behavior of the EFT approximation has no resemblance to the high-energy behavior of the resonance model, regardless of unitarity projection. Experimentally, for a weakly-coupled resonance a resonance search in VBS based on such a simplified model as signal model seems to be much more promising than a search for deviations in terms of SMEFT Wilson coefficients.

We conclude that including resonance models in the description allows us to smoothly interpolate between weakly and strongly interacting models. This interpolation may leave the applicability range of perturbative expansions, but does not require to deal with unphysical behavior as a calculational artefact.

## Implications for LHC analyses and conclusions

The ATLAS and CMS experiments have analyzed the early-stage LHC data with respect to the sensitivity to VBS parameters. Table 3 summarizes published results, expressed in terms of the unmodified SMEFT parameterization with dimension-eight operators included.

**Table 3:** Observed limits of ATLAS and CMS of complete LHC data at $\sqrt{s}=8\, \mathrm {TeV}$ and current observed limits of CMS at $\sqrt{s}=13\, \mathrm {TeV}$ using the naive EFT model and the T-matrix model. The last column shows the limits in natural reweighting of field strength tensors: $\mathbf {W}^{\mu \nu }\rightarrow \mathrm {i}g \mathbf {W}^{\mu \nu }$, $\mathbf {B}^{\mu \nu }\rightarrow \mathrm {i}g^\prime \mathbf {B}^{\mu \nu }$

Coefficient [TeV$^{-4}$]	CMS&ATLAS [56] 8 TeV, EFT	ATLAS [57, 58] 8 TeV, T-matrix	CMS [59, 60] 13 TeV, EFT	CMS reweighted 13 TeV, EFT
$f_{S_0}/\varLambda ^4$	[$-$ 38, 40]		[$-$ 7.7, 7.7]	[$-$ 7.7, 7.7]
$f_{S_1}/\varLambda ^4$	[$-$ 118,120]		[$-$ 21.6, 21.8]	[$-$ 21.6, 21.8]
$F_{S_0}$	[$-$ 70, 70]	[$-$ 104, 130]		
$F_{S_1}$	[$-$ 118,120]	[$-$ 122, 144]		
$f_{M_0}/\varLambda ^4$	[$-$ 18, 18]		[$-$ 6.0, 5.9]	[$-$ 13.8, 14.1]
$f_{M_1}/\varLambda ^4$	[$-$ 44, 47]		[$-$ 8.7, 9.1]	[$-$ 21.4, 20.4]
$f_{M_6}/\varLambda ^4$	[$-$ 65, 63]		[$-$ 11.9, 11.8]	[$-$ 27.7, 27.9]
$f_{M_7}/\varLambda ^4$	[$-$ 70, 66]		[$-$ 13.3, 12.9]	[$-$ 30.3, 31.2]
$f_{T_0}/\varLambda ^4$	[$-$ 4.2, 4.6]		[$-$ 0.46, 0.44]	[$-$ 2.53, 2.42]
$f_{T_1}/\varLambda ^4$	[1.9, 2.2]		[$-$ 0.28, 0.31]	[$-$ 1.54, 1.71]
$f_{T_2}/\varLambda ^4$	[$-$ 5.2, 6.4]		[$-$ 0.89, 1.02]	[$-$ 4.9, 5.6]
$f_{T_9}/\varLambda ^4$	[$-$ 6.9, 6.9]		[$-$ 1.8, 1.8]	[$-$ 7.5, 7.5]

In view of the results presented in the preceding sections, we have to discuss the physical relevance of the published exclusion bounds. In principle, the SMEFT approach provides a well-defined framework. However, our findings confirm the expectation that the SMEFT expansion, applied to VBS as a LHC process, does not provide a systematic expansion or meaningful description of the complete data set. For nonvanishing dimension-eight coefficients, the amplitudes rise steeply with energy, such that a problem invariably arises within the accessible kinematic range. This happens for *any* set of parameter values, unless the dimension-eight coefficients are so small that the prediction remains entirely indistinguishable from the SM.

The measurements acquire a physical interpretation only within the context of a unitary model. For instance, we may apply a straightforward T-matrix projection to the naive extrapolation and thus consider a unitary simplified model that is smoothly matched to the low-energy SMEFT, depending on the same parameters that in the low-energy act as dimension-eight operator coefficients. We find that the sensitivity of this unitary model to the free parameters is much weaker than the naive calculation would suggest, likely by an order of magnitude. Since the minimal T-matrix projection interpolates the low-energy behavior with asymptotic saturation of the elastic channel, this particular projection provides us with the ultimate limitation to the achievable parameter sensitivity.

We conclude that any such description or theoretical prediction of non-SM behavior has to depart from the model-independent paradigm. Otherwise, data analysis has to artificially remove kinematical regions from the data sample, losing valuable information. A well-defined universal but model-dependent parameterization is certainly possible, however, without losing contact to the SMEFT as a systematic description of the low-energy region.

In this work, we have demonstrated the construction of unitary projections that yield usable simplified models for otherwise unknown new physics. Extending previous work, we have included transverse vector-boson polarization modes together with final-state Higgs bosons in the completed framework. None of our models is UV complete or otherwise meaningful as a prediction. However, for the purpose of estimating the prospects for future measurements in quantitative terms, such a set of simplified models becomes a useful tool. Applying the direct T-matrix projection to the straightforward extrapolation of the SMEFT amplitudes with dimension-eight operators, we obtain a natural interpolation between the low-energy range which is well understood, and high-energy amplitudes which saturate the unitarity limits. This sets the scale for more refined models, such as the model of a singlet scalar coupled to transverse gauge bosons which we also have considered in some detail. In essence, we obtain parameter-dependent upper limits for event rates of all processes for all energy ranges, which refined models have to respect.

The lesson to be learned from such results is twofold. Firstly, we read off the range of event rates and distributions that we can possibly expect from LHC experiments, for any underlying model. This range can only be exceeded if some natural, basic assumptions are violated by Nature. More precisely, violations of the assumptions would point to (a) fermions directly involved in new (strong) Higgs-sector interactions, or (b) gauge symmetry being just a low-energy accident, or (c) four-dimensional quantum field theory becoming invalid. Either scenario appears to be unlikely given the success of the SM in describing low-energy data, in particular in the flavor sector. For this reason, we believe that the quantitative results obtained within the framework of unitary simplified models reliably exhaust the range that can be expected from real data.

Secondly, the framework of unitary projection, now extended to transverse polarizations, enables any theoretical idea or model of the Higgs sector as a viable model for Monte-Carlo simulations, i.e., the projection satisfies the applicable unitarity constraints, correctly couples to fermionic currents, and the collider environment is described in consistency with the analogous SM calculation. In short, the model can be compared to data without further approximations or simplifications. The downside is that for VBS processes, there is no usable model-independent framework, and any study has to agree on a particular model class and assumptions for interpreting the results. Furthermore, the arbitrariness in the parameterization mandates the inclusion of all quantum-number combinations and global fits, which would greatly benefit from a larger set of observables such as can be obtained at high-energy lepton colliders supplementing the LHC.

Finally, we add a remark on Higgs pair-production. This final state has received particular attention since it is sensitive to the triple-Higgs coupling and thus to the Higgs potential. Higgs pairs can result from gluon or massive vector-boson fusion. The latter channel has the particular feature of extra taggable forward jets. In a generic EFT description, the Higgs pair-production process in VBF receives various contributions that can be attributed to higher-dimensional operators, and the Higgs potential correction is only one of those. Furthermore, our results show that dimension-eight operators can drastically enhance the Higgs pair-production rate by three orders of magnitude before unitarity limits set in. Since there is no reason to expect the operator series expansion to stop at dimension six, we are forced to argue that any analysis of Higgs pair-production data that confines itself to a truncated expansion has to be taken with a grain of salt. On the other hand, linear gauge invariance relates anomalous effects in Higgs pair-production to anomalous effects in VBS at the same order. Future LHC Higgs pair-production analyses thus should correlate all accessible boson-production channels. The interpretation, however, will rely on model-dependent approaches such as the one that we present in this paper.

## Figures and Tables

**Fig. 6:**
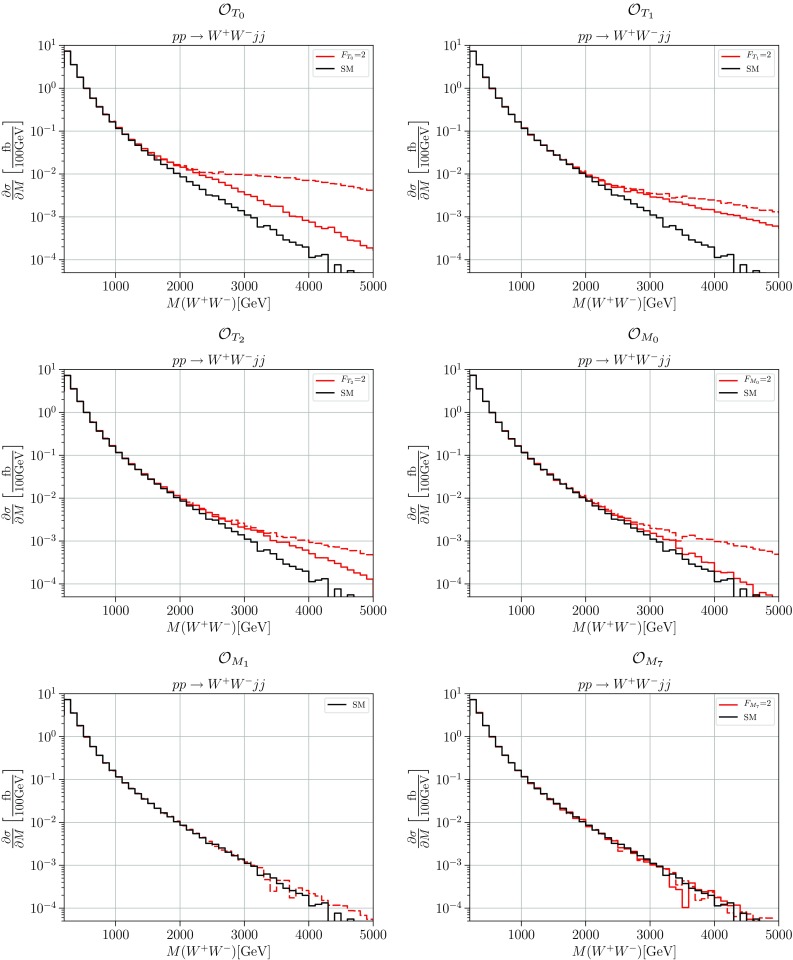
The plot shows the differential cross section as a function of the invariant mass $m_{VV}$ of the two colliding vector bosons. The solid black line is the standard model and the colored lines are the contributions of ${\mathcal {O}}_{T_{0, 1, 2}}$ and ${\mathcal {O}}_{M_{{0, 1, 7}}}$ (dashed: naive EFT, solid: unitarized model). Cuts: $M_{jj} > 500$ GeV; $\varDelta \eta _{jj} > 2.4$; $p^j_T > 20$ GeV; $|\eta _j| > 4.5$

**Fig. 7:**
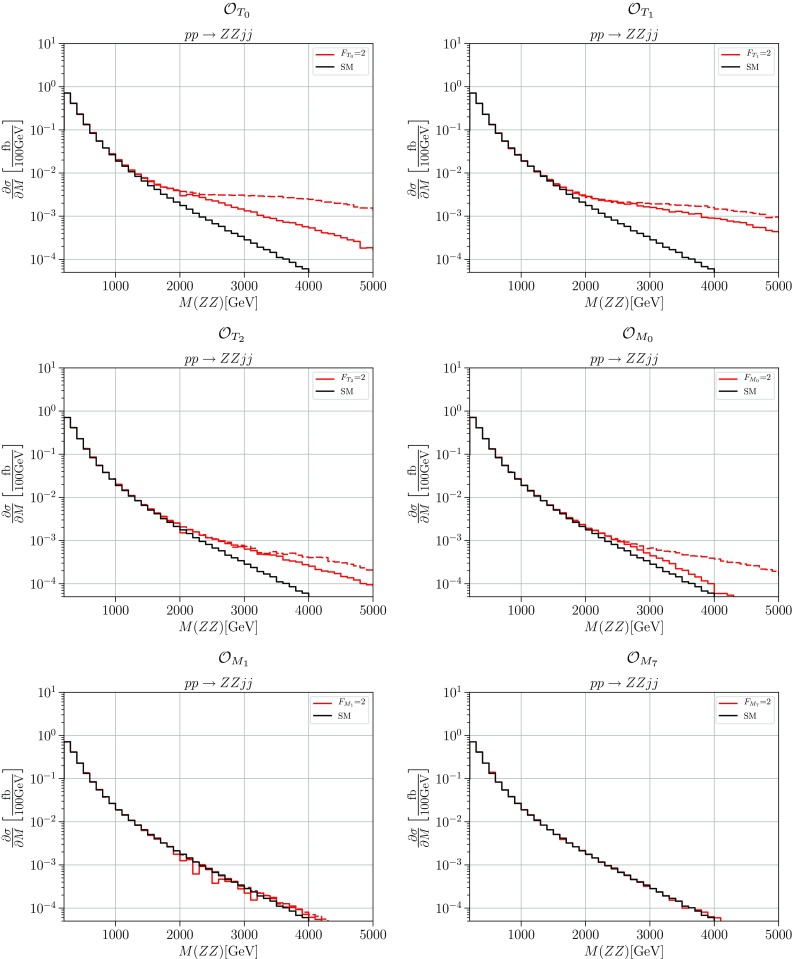
The plot shows the differential cross section as a function of the invariant mass $m_{VV}$ of the two colliding vector bosons. The solid black line is the standard model and the colored lines are the contributions of ${\mathcal {O}}_{T_{0, 1, 2}}$ and ${\mathcal {O}}_{M_{0,1,7}}$ (dashed: naive EFT, solid: unitarized model). Cuts: $M_{jj} > 500$ GeV; $\varDelta \eta _{jj} > 2.4$; $p^j_T > 20$ GeV; $|\eta _j| > 4.5$

**Fig. 8:**
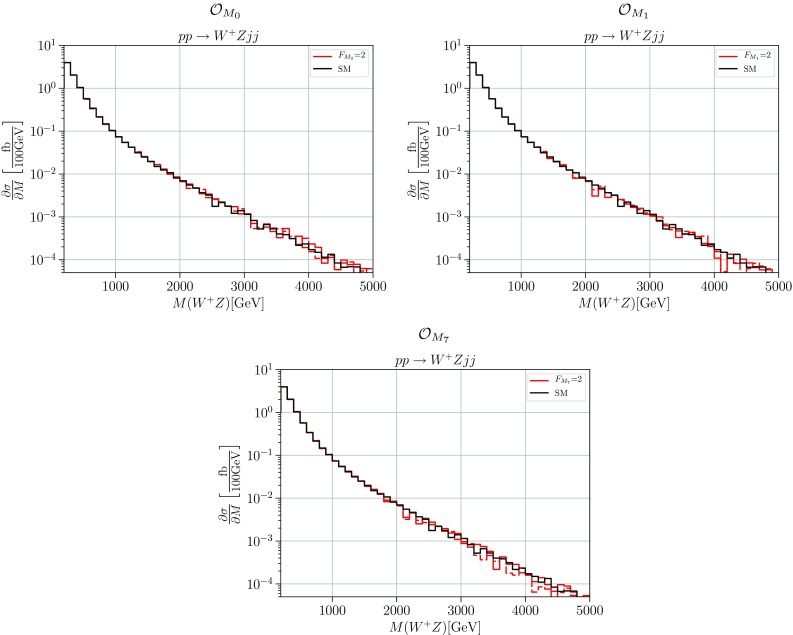
The plot shows the differential cross section as a function of the invariant mass $m_{VV}$ of the two colliding vector bosons. The solid black line is the standard model and the colored lines are the contributions of ${\mathcal {O}}_{M_{0,1,7}}$ (dashed: naive EFT, solid: unitarized model). Cuts: $M_{jj} > 500$ GeV; $\varDelta \eta _{jj} > 2.4$; $p^j_T > 20$ GeV; $|\eta _j| > 4.5$

## A Isospin-spin amplitudes

In this section we collect the different isospin-spin eigenamplitudes for the different combinations of helicities of the electroweak gauge bosons according to decomposition into Wigner functions in Eq. 14. As explained in the last paragraph of Sec. 2, we take here only the Wilson coefficients of those transversal and mixed operators ${\mathcal {L}}_{T_i}$ and ${\mathcal {L}}_{M_i}$, respectively, with indices $i=0,1,2$ into account for which there is custodial $SU(2)_C$ conserved.

Already in Ref. [9] it was shown that the kinematic functions for the unitarized amplitudes for resonances are not simple powers of *s*, but contain logarithms and pole-like rational functions of *s* in general. For the isospin-spin amplitudes in the case of a isoscalar scalar resonance in Table 4, we define the following kinematic functions:

23 $$\begin{aligned} X(s,m)&=\frac{3s^2}{s-m^2} \end{aligned}$$

24 $$\begin{aligned} {\mathcal {S}}_0(s,m)&=2m^2+2\frac{m^4}{s} \log \left( \frac{m^2}{s+m^2} \right) -s \end{aligned}$$

25 $$\begin{aligned} {\mathcal {S}}_1(s,m)&=4\frac{m^4}{s}+6m^4\left( 2m^2+s\right) \log \left( \frac{m^2}{s+m^2} \right) +\frac{s}{3} \end{aligned}$$

26 $$\begin{aligned} {\mathcal {S}}_2(s,m)&=6\frac{m^4}{s^2}\left( 2m^2+s\right) \nonumber \\&\qquad \quad + 2\frac{m^4}{s^3} \left( 6m^4+6m^2 s+s^2\right) \log \left( \frac{m^2}{s+m^2} \right) \end{aligned}$$

27 $$\begin{aligned} {\tilde{{\mathcal {S}}}_2}(s,m)&=\frac{m^2}{3}-\frac{m^4}{2s} +\frac{m^6}{s^2} + \frac{m^8}{s^3}\log \left( \frac{m^2}{s+m^2} \right) -\frac{s}{4} \end{aligned}$$

**Table 4:** Coefficients *c* of the isospin spin amplitudes calculated with Eq. (14) for the isoscalar scalar resonance ${\mathcal {L}}_{\sigma W}$ depending on the helicity of the incoming and outgoing particles. The isospin spin amplitudes are given by $A_{ij}(s; {\varvec{\lambda }})=c g^4 s^2$

i	j						
	0	1	2	${\varvec{\lambda }}$			
0	*X*(*s*, *m*)	0	0	$+$	$+$	$+$	$+$
	0	0	${\tilde{{\mathcal {S}}}_2}(s,m)$	$+$	−	$+$	−
	0	0	${\tilde{{\mathcal {S}}}_2}(s,m)$	$+$	−	−	$+$
	$X(s,m)+{\mathcal {S}}_0(s,m)$	0	${\mathcal {S}}_2(s,m)$	$+$	$+$	−	−
1	0	0	0	$+$	$+$	$+$	$+$
	0	0	$-{\tilde{{\mathcal {S}}}_2}(s,m)$	$+$	−	$+$	−
	0	0	${\tilde{{\mathcal {S}}}_2}(s,m)$	$+$	$+$	$+$	$+$
	0	${\mathcal {S}}_1(s,m)$	0	$+$	$+$	−	−
2	0	0	0	$+$	$+$	$+$	$+$
	0	0	${\tilde{{\mathcal {S}}}_2}(s,m)$	$+$	−	$+$	−
	0	0	${\tilde{{\mathcal {S}}}_2}(s,m)$	$+$	−	−	$+$
	${\mathcal {S}}_0(s,m)$	0	${\mathcal {S}}_2(s,m)$	$+$	$+$	−	−

## B Additional numerical results

In this section, we display results for the invariant-mass distribution of the LHC processes $pp\rightarrow W^+W^-jj$, *ZZjj*, and $W^+Zjj$ in Figs. 6, 7, 8 which supplement the results for the $W^+W^+$ and *HH* channels in the main text. For all processes, we present the SM distribution together with the corresponding distribution of the continuum simplified model, one free parameter varied at a time, with a universal parameter value of $2\;\text {TeV}^{-4}$.

## Footnotes

1 Heavy fermions (*t*, *b*, $\tau$) should be taken into account in this context, but we do not study the corresponding final states in this paper.

2 There are two directions in the dimension-six operator space which in our context correspond to $(J_H^{(2)})^3$ and $J_H^{(2)}J_H^{(4)}$. An unambiguous determination of the coefficients requires measurements of Higgs-pair production and the Higgs total width, respectively.

3 WHIZARD is a multi-purpose event generator which ships with its a tree-level matrix element generator [27, 28]. It uses the color-flow formalism for QCD [29], and comes with its own parton shower implementations [30]. While it allows to simulate almost arbitrary BSM interactions (cf. e.g. the SUSY implementation [31]) via its FeynRules interface [32], WHIZARD has been also successfully extended towards next-to-leading order and matched to gluon and photon resummation [33–39].

## References

[1] G. Aad et al., [ATLAS Collaboration], Observation of a new particle in the search for the Standard Model Higgs boson with the ATLAS detector at the LHC. Phys. Lett. B **716**, 1 (2012). 10.1016/j.physletb.2012.08.020. arXiv:1207.7214 [hep-ex]

[2] S. Chatrchyan et al., [CMS Collaboration], Observation of a new boson at a mass of 125 GeV with the CMS experiment at the LHC. Phys. Lett. B **716**, 30 (2012). 10.1016/j.physletb.2012.08.021. arXiv:1207.7235 [hep-ex]

[3] Buchmüller W, Wyler D (1986). Effective lagrangian analysis of new interactions and flavor conservation. Nucl. Phys. B.

[4] Hagiwara K, Ishihara S, Szalapski R, Zeppenfeld D (1992). Low-energy constraints on electroweak three gauge boson couplings. Phys. Lett. B.

[5] Hagiwara K, Ishihara S, Szalapski R, Zeppenfeld D (1993). Low-energy effects of new interactions in the electroweak boson sector. Phys. Rev. D.

[6] Grzadkowski B, Iskrzynski M, Misiak M, Rosiek J (2010). Dimension-six terms in the standard model Lagrangian. JHEP.

[7] Kilian W, Ohl T, Reuter J, Sekulla M (2015). High-energy vector boson scattering after the Higgs discovery. Phys. Rev. D.

[8] Kalinowski J, Kozw P, Pokorski S, Rosiek J, Szleper M, Tkaczyk S (2018). Eur. Phys. J. C.

[9] Kilian W, Ohl T, Reuter J, Sekulla M (2016). Resonances at the LHC beyond the Higgs boson: the scalar/tensor case. Phys. Rev. D.

[10] Fleper C, Kilian W, Reuter J, Sekulla M (2017). Scattering of W and Z bosons at high-energy lepton colliders. Eur. Phys. J. C.

[11] Jäger B, Oleari C, Zeppenfeld D (2009). Next-to-leading order QCD corrections to $$W^+W^+jj$$ and $$W^-W^-jj$$ production via weak-boson fusion. Phys. Rev. D.

[12] Melia T, Melnikov K, Röntsch R, Zanderighi G (2010). Next-to-leading order QCD predictions for $$W^+W^+jj$$ production at the LHC. JHEP.

[13] Melia T, Melnikov K, Röntsch R, Zanderighi G (2011). NLO QCD corrections for $$W^+W^-$$ pair production in association with two jets at Hadron Colliders. Phys. Rev. D.

[14] Jäger B, Zanderighi G (2011). NLO corrections to electroweak and QCD production of $$W^+W^+$$ plus two jets in the POWHEGBOX. JHEP.

[15] J. Baglio et al., Release Note—VBFNLO 2.7.0, arXiv:1404.3940 [hep-ph]

[16] Biedermann B, Denner A, Pellen M (2017). Large electroweak corrections to vector-boson scattering at the Large Hadron Collider. Phys. Rev. Lett..

[17] Biedermann B, Denner A, Pellen M (2017). Complete NLO corrections to W$$^{+}$$W$$^{+}$$ scattering and its irreducible background at the LHC. JHEP.

[18] Ballestrero A. (2018). Precise predictions for same-sign W-boson scattering at the LHC.. Eur. Phys. J. C.

[19] Eboli OJP, Gonzalez-Garcia MC, Mizukoshi JK (2006). $$p p \rightarrow j j e^\pm \mu ^\pm \nu \nu $$ and $$j j e^\pm \mu ^\mp \nu \nu $$ at $${\cal{O}}(\alpha _{em}^6)$$ and $${\cal{O}}(\alpha _{em}^4 \alpha _s^2)$$ for the study of the quartic electroweak gauge boson vertex at CERN LHC. Phys. Rev. D.

[20] Degrande C (2014). A basis of dimension-eight operators for anomalous neutral triple gauge boson interactions. JHEP.

[21] M. Baak et al., Working group report: precision study of electroweak interactions. in *Proceedings, 2013 Community Summer Study on the Future of U.S. Particle Physics: Snowmass on the Mississippi (CSS2013): Minneapolis, MN, USA, July 29-August 6, 2013.* arXiv:1310.6708 [hep-ph]

[22] Perez G, Sekulla M, Zeppenfeld D (2018). Eur. Phys. J. C.

[23] Alboteanu A, Kilian W, Reuter J (2008). Resonances and unitarity in weak boson scattering at the LHC. JHEP.

[24] M. Sekulla, Anomalous couplings, resonances and unitarity in vector boson scattering, Ph.D. thesis, University of Siegen (2015)

[25] E.P. Wigner, *Gruppentheorie und ihre Anwendungen auf die Quantenmechanik der Atomspektren*. (Vieweg Vlg, Braunschweig, 1931)

[26] Kilian W, Ohl T, Reuter J (2011). WHIZARD: simulating multi-particle processes at LHC and ILC. Eur. Phys. J. C.

[27] M. Moretti, T. Ohl, J. Reuter, O’Mega: an optimizing matrix element generator, in *2nd ECFA/DESY Study 1998-2001* 1981–2009. arXiv:hep-ph/0102195

[28] Chokoufe Nejad B, Ohl T, Reuter J (2015). Simple, parallel virtual machines for extreme computations. Comput. Phys. Commun.

[29] Kilian W, Ohl T, Reuter J, Speckner C (2012). QCD in the color-flow representation. JHEP.

[30] Kilian W, Reuter J, Schmidt S, Wiesler D (2012). An analytic initial-state parton shower. JHEP.

[31] Ohl T, Reuter J (2003). Clockwork SUSY: supersymmetric ward and Slavnov–Taylor identities at work in Green’s functions and scattering amplitudes. Eur. Phys. J. C.

[32] Christensen ND, Duhr C, Fuks B, Reuter J, Speckner C (2012). Introducing an interface between WHIZARD and FeynRules. Eur. Phys. J. C.

[33] Bach F, Nejad BC, Hoang A, Kilian W, Reuter J, Stahlhofen M, Teubner T, Weiss C (2018). Fully-differential top-pair production at a Lepton Collider: from threshold to continuum. JHEP.

[34] Chokoufé Nejad B, Kilian W, Lindert JM, Pozzorini S, Reuter J, Weiss C (2016). NLO QCD predictions for off-shell $$ t{\overline{t}} $$ and $$ t{\overline{t}}H $$ production and decay at a linear collider. JHEP.

[35] B. Chokoufe Nejad, W. Kilian, J. Reuter, C. Weiss, Matching NLO QCD corrections in WHIZARD with the POWHEG scheme, PoS EPS **-HEP2015**, 317 (2015). arXiv:1510.02739 [hep-ph]

[36] C. Weiss, B. Chokoufe Nejad, W. Kilian, J. Reuter, Automated NLO QCD corrections with WHIZARD, PoS EPS **-HEP2015**, 466 (2015). arXiv:1510.02666 [hep-ph]

[37] Greiner N, Guffanti A, Reiter T, Reuter J (2011). NLO QCD corrections to the production of two bottom-antibottom pairs at the LHC. Phys. Rev. Lett..

[38] Binoth T, Greiner N, Guffanti A, Reuter J, Guillet J-P, Reiter T (2010). Next-to-leading order QCD corrections to $$pp \rightarrow b {\bar{b}} b {\bar{b}} + X$$ at the LHC: the quark induced case. Phys. Lett. B.

[39] Kilian W, Reuter J, Robens T (2006). NLO event generation for chargino production at the ILC. Eur. Phys. J. C.

[40] Buttazzo D, Greljo A, Marzocca D (2016). Knocking on new physics door with a scalar resonance. Eur. Phys. J. C.

[41] Giddings SB, Zhang H (2016). Kaluza–Klein graviton phenomenology for warped compactifications, and the 750 GeV diphoton excess. Phys. Rev. D.

[42] Han C, Lee HM, Park M, Sanz V (2016). The diphoton resonance as a gravity mediator of dark matter. Phys. Lett. B.

[43] Randall L, Sundrum R (1999). A large mass hierarchy from a small extra dimension. Phys. Rev. Lett..

[44] Arkani-Hamed N, Dimopoulos S, Dvali GR (1998). The hierarchy problem and new dimensions at a millimeter. Phys. Lett. B.

[45] Geng CQ, Huang D (2016). Note on spin-2 particle interpretation of the 750 GeV diphoton excess. Phys. Rev. D.

[46] Pasechnik R, Beylin V, Kuksa V, Vereshkov G (2013). Chiral-symmetric technicolor with standard model Higgs boson. Phys. Rev. D.

[47] Yoon YW, Cheung K, Kang SK, Song J (2017). Radiative decays of a singlet scalar boson through vectorlike quarks. Phys. Rev. D.

[48] Bauer M, Hörner C, Neubert M (2016). Diphoton resonance from a warped extra dimension. JHEP.

[49] Franceschini R (2016). What is the $$\gamma \gamma $$ resonance at 750 GeV?. JHEP.

[50] Gupta RS, Jäger S, Kats Y, Perez G, Stamou E (2016). Interpreting a 750 GeV diphoton resonance. JHEP.

[51] Kim JS, Reuter J, Rolbiecki K, Ruiz de Austri R (2016). A resonance without resonance: scrutinizing the diphoton excess at 750 GeV. Phys. Lett. B.

[52] Delgado RL, Dobado A, Espriu D, Garcia-Garcia C, Herrero MJ, Marcano X, Sanz-Cillero JJ (2017). Production of vector resonances at the LHC via WZ-scattering: a unitarized EChL analysis. JHEP.

[53] Arkani-Hamed N, Cohen AG, Georgi H (2001). Electroweak symmetry breaking from dimensional deconstruction. Phys. Lett. B.

[54] Arkani-Hamed N, Cohen AG, Katz E, Nelson AE (2002). The Littlest Higgs. JHEP.

[55] Kilian W, Reuter J (2004). The Low-energy structure of little Higgs models. Phys. Rev. D.

[56] Green DR, Meade P, Pleier MA (2017). Multiboson interactions at the LHC. Rev. Mod. Phys..

[57] M. Aaboud et al., [ATLAS Collaboration], Search for anomalous electroweak production of $$WW/WZ$$ in association with a high-mass dijet system in $$pp$$ collisions at $$\sqrt{s}=8$$ TeV with the ATLAS detector. Phys. Rev. D **95**(3), 032001 (2017). 10.1103/PhysRevD.95.032001. arXiv:1609.05122 [hep-ex]

[58] Sekulla M, Kilian W, Ohl T, Reuter J (2016). Effective field theory and unitarity in vector boson scattering. PoS LHCP.

[59] A.M. Sirunyan et al., [CMS Collaboration], Observation of electroweak production of same-sign W boson pairs in the two jet and two same-sign lepton final state in proton-proton collisions at $$\sqrt{s} = $$ 13 TeV. arXiv:1709.05822 [hep-ex] 10.1103/PhysRevLett.120.081801 29542998

[60] A.M. Sirunyan et al., [CMS Collaboration], Measurement of vector boson scattering and constraints on anomalous quartic couplings from events with four leptons and two jets in protonproton collisions at $$\sqrt{s}=$$ 13 TeV. Phys. Lett. B **774**, 682 (2017). 10.1016/j.physletb.2017.10.020

